# Pioglitazone-Mediated Attenuation of Experimental Colitis Relies on Cleaving of Annexin A1 Released by Macrophages

**DOI:** 10.3389/fphar.2020.591561

**Published:** 2020-12-21

**Authors:** Gustavo Henrique Oliveira da Rocha, Marina de Paula-Silva, Milena Fronza Broering, Pablo Rhasan dos Santos Scharf, Larissa Satiko Alcântara Sekimoto Matsuyama, Silvya Stuchi Maria-Engler, Sandra Helena Poliselli Farsky

**Affiliations:** Department of Clinical and Toxicological Analyses, Faculty of Pharmaceutical Sciences, University of São Paulo, São Paulo, Brazil

**Keywords:** Inflammatory bowel disease, experimental colitis, pioglitazone, macrophage, annexin A1

## Abstract

Ulcerative colitis and Crohn’s disease are chronic inflammatory bowel diseases (IBDs) which burden health systems worldwide; available pharmacological therapies are limited and cost-intensive. Use of peroxisome proliferator activated-receptor γ (PPARγ) ligands for IBD treatment, while promising, lacks solid evidences to ensure its efficacy. Annexin A1 (AnxA1), a glucocorticoid-modulated anti-inflammatory protein, plays a key role on IBD control and is a potential biomarker of IBD progression. We here investigated whether effects of pioglitazone, a PPARγ ligand, rely on AnxA1 actions to modulate IBD inflammation. Experimental colitis was evoked by 2% dextran sodium sulfate (DSS) in AnxA1 knockout (AnxA1^−/−^) or wild type (WT) C57BL/6 mice. Clinical and histological parameters were more severe for AnxA^−/−^ than WT mice, and 10 mg/kg pioglitazone treatment attenuated disease parameters in WT mice only. AnxA1 expression was increased in tissue sections of diseased WT mice, correlating positively with presence of CD68^+^ macrophages. Metalloproteinase-9 (MMP-9) and inactive 33 kDa AnxA1 levels were increased in the colon of diseased WT mice, which were reduced by pioglitazone treatment. Cytokine secretion, reactive oxygen species generation and MMP-9 expression caused by lipopolysaccharide (LPS) treatment in AnxA1-expressing RAW 264.7 macrophages were reduced by pioglitazone treatment, effects not detected in AnxA1 knockdown macrophages*.* LPS-mediated increase of AnxA1 cleaving in RAW 264.7 macrophages was also attenuated by pioglitazone treatment. Finally, pioglitazone treatment increased extracellular signal-regulated kinase (ERK) phosphorylation in AnxA1-expressing RAW 264.7 macrophages, but not in AnxA1-knockdown macrophages. Thus, our data highlight AnxA1 as a crucial factor for the therapeutic actions of pioglitazone on IBDs.

## Introduction

Ulcerative colitis and Crohn's disease comprise inflammatory bowel diseases (IBDs), which are widespread and pose burdens to healthcare systems worldwide (Mak et al., 2020). In 2016 in the United States alone, the prevalence of both maladies combined was last reported to be of one for every 209 people, and incidence increases steadily each year (Luther and Dave, 2020); also, the estimated loss of earnings due to IBDs were calculated as $31 billion in the same year (Xu et al., 2018). Patients suffer from diarrhea, anemia, fatigue and severe gut pain, among other symptoms, which become more intense during acute episodes, causing them to lose workdays and drastically reducing quality of life (Xu et al., 2018). Pharmacological options to treat these diseases are limited, as traditionally used drugs address only the major symptoms of inflammatory bowel diseases and fail to conduct patients to complete remission (Duijvestein et al., 2018). Biological therapies involving anti-TNFα antibodies, such as infliximab, while proven successful and better than conventional drugs, do not cause any response in about a third of patients and are very cost-intensive (Hossain et al., 2020). There is also a trend nowadays for new IBD therapies to aim for objective targets in an individualized manner rather than following pre-determined therapy paradigms (e.g., aim for mucosal healing rather than “overall remission”), and thus the understanding of new IBD biomarkers and of how new or already existing drugs work and with which other molecules they interact on intestinal tissue becomes a necessity (Im et al., 2018; Ho et al., 2020).

Annexin A1 (AnxA1), a 37 kDa protein modulated by the actions of glucocorticoids is one such molecule currently assessed as a potential target for therapy of not only IBDs, but of other inflammatory chronic diseases as well, playing roles on the inflammatory modulation of diseases such as asthma, type-2 diabetes, cystic fibrosis, rheumatoid arthritis, among others (Patel et al., 2012; Perretti and D’acquisto, 2009; Perucci et al., 2017; Sheikh and Solito, 2018). It is expressed by epithelial cells and monocytes, neutrophils and macrophages to a great extent (Perretti and D’acquisto, 2009). In IBD animal models, AnxA1 and its N-terminal mimetic peptides are known to attenuate disease progression and promote epithelial repair (Babbin et al., 2008; Ouyang et al., 2012; Zou et al., 2016), and when lacking halts disease remission caused by treatment with infliximab (de Paula-Silva et al., 2016). AnxA1 expression is increased in intestinal tissue of ulcerative colitis patients while decreased in Crohn's disease patients, evidencing these diseases deregulate AnxA1 actions leading to compromised anti-inflammatory responses (Vong et al., 2012; Sena et al., 2013). It has been reported AnxA1 also plays a role on healing of damaged intestinal epithelium on murine IBD models, and that its expression is correlated with better prognosis on Crohn's disease patients (Leoni et al., 2015; Reischl et al., 2020).

While seemingly a beneficial role player on downmodulating inflammation, AnxA1 can be cleaved into smaller 33 kDa fragments which are believed to be non-functional and even lead to pro-inflammatory effects, such as increased neutrophil transmigration via endothelium (Williams et al., 2010; Sugimoto et al., 2016); while this event has not yet been described in neither IBD patients nor in experimental colits animal models, it has been evidenced cleaved AnxA1 correlates with increased inflammatory damage in animal pleurisy models and in neutrophils recruited from animals treated with LPS (Vago et al., 2012; Vago et al., 2016).

In the search for new pharmacological targets to treat IBDs, in the same vein as AnxA1, peroxisome proliferator activated γ (PPARγ) has been explored as a potential transcription factor linked to modulation of IBD, and it has been evidenced its impaired expression is linked to disease progression in humans (Yamamoto-Furusho et al., 2011; Dou et al., 2015). PPARγ ligands such as thiazolidinediones have emerged as potential new anti-inflammatory candidates for IBD therapy, and first reviews on the efficacy of PPARγ ligands for treatment of IBDs date back 20 years ago (Wada et al., 2001). While traditionally used for treatment of diabetes, such ligands have been explored as anti-inflammatories in the past few years and shown as promising treatment options for several chronic inflammatory diseases, especially those that affect the central nervous system, such as Alzheimer’s disease, Parkinson’s disease and multiple sclerosis (Galimberti and Scarpini, 2017; Bonato et al., 2018). Pioglitazone, one such PPARγ ligand, is also effective in attenuating inflammation in alcoholic-induced cirrhosis livers and reducing specific histologic lesions in cancerous lungs of smokers (Chongmelaxme et al., 2019; Keith et al., 2019). On macrophages, pioglitazone is reported to induce M2 polarization, attenuate TNFα and IL-1ß expressions, reduce actions of CXCL1 and CCL2 and increase the expression of IL-10 and TGF-ß (Fernandez-Boyanapalli et al., 2010; Ahmadian et al., 2013; Pisanu et al., 2014). Most importantly, however, is pioglitazone role on attenuating the progression of IBDs, as demonstrated by experimental animal models, where treatment of animals induced to development of IBD causes prevention of weight loss, mucosal healing and epithelium restructuration via increase of ZO-1 and claudin-5; pioglitazone also attenuates overall inflammation by reducing NF-kB activation, secretion of cytokines (such as IL-2, TNFα and IL-17) and myeloperoxidase activity (Takagi et al., 2002; Takaki et al., 2006; Da Silva et al., 2018; El Awdan and Mostafa, 2018; Huang et al., 2020).

Even though several animal studies evidence the beneficial role of pioglitazone on treatment and remission of IBDs, the few studies involving pioglitazone and other PPARγ ligands on humans, while conducted to some success, at the end have failed to advance to further stages of clinical trials or relied only on *in silico* approaches (Liang and Ouyang, 2008; Abedi et al., 2015). AnxA1 expression is associated with progression of inflammation in both IBD models and in patients, and previous findings from our group evidenced AnxA1 plays a role on PPARγ activation and actions (da Rocha et al., 2019). Therefore, we aimed to investigate whether AnxA1 could play a role on pioglitazone-mediated resolution of inflammation in an IBD model in order to elucidate how pioglitazone ameliorates disease progression.

## Material and Methods

### *In Vivo* Experimentation

#### Animals Used

Wild type (WT) or knockout annexin A1 (AnxA1^−/−^) male C57Bl/6 mice were obtained from the Center for Development of Experimental Models for Biology and Medicine, Federal University of São Paulo (CEDEME/UNIFESP). Animals were kept under 12 h light/dark cycles at 25°C and were given water and feed *ad libitum*. Experiments were carried in accordance with Ethical Principles for Animal Experimentation (COBEA) and were approved under protocol no. 577 by the Ethics Committee on Animal Use of the Faculty of Pharmaceutical Sciences of the University of São Paulo (CEUA/FCF/USP).

#### Experimental Colitis and Pharmacological Treatment

Acute colitis was induced by ingestion of dextran sodium sulfate (DSS) 40 kDa (Dextran Products Limited, Scarborough, ON, Canada) administered to the drinking water of mice. Throughout a period of 6 days mice consumed water containing 2% DSS 40 kDa, replaced every 2 days so DSS consumption would be homogeneous (de Paula-Silva et al., 2016).

WT and AnxA1^−/−^ mice were divided in the following groups: 1) Control (driking water with no other additives), 2) DSS and 3) DSS + pioglitazone. All mice received daily injections of either pioglitazone (Sigma-Aldrich, St. Louis, MO, United States) at a dose of 10 mg/kg or its vehicle (0.9% sterile saline +0.5% carboxymethylcellulose, Sigma-Aldrich, St. Louis, MO, United States). Pioglitazone was prepared in vehicle suspension and sonicated in a sonic bath USC 800^®^ (Unique, Indaiatuba, SP, Brazil) for 30 min. A volume of 200 µl of the pioglitazone suspension was injected intraperitoneally into mice. The experimental protocol was based on previous works from our research group (Santin et al., 2013; de Paula-Silva et al., 2016). A graphical representation of the experimental protocol is described in Supplementary Material (Supplementary Figure S1).

#### Evaluation of Clinical Parameters

Clinical parameters of animals were verified daily during induction and progression of colitis. Animals were weighted; consistency of feces was determined by collecting a piece of stool and pressing it with forceps in order to verify its softness/hardness; rectal blood was assessed on feces tested for occult blood. For detection of occult blood, a piece of stool was dispersed into 150 µl of 0.9% saline solution alongside 150 µl of a benzidine (Sigma-Aldrich, St. Louis, MO, United States) and hydrogen peroxide (Synth, Diadema, SP, Brazil) solution; the resulting greenish solution was scored according to its intensity. All mentioned parameters were scored from 0 to 4 as follows (0) weight loss inferior to 1%, solid feces, absence of occult blood (Mak et al., 2020); weight loss ranging from 1 to 5%, slightly softened feces, small amount of occult blood (Luther and Dave, 2020); weight loss ranging from 5 to 10%, softened feces, moderate amount of occult blood (Xu et al., 2018); weight loss ranging from 10 to 20%, pastry feces, considerable amount of occult blood (Duijvestein et al., 2018); weight loss superior to 20%, liquid feces, high amount of occult blood. Scores were summed at each day, resulting in the disease Activity Index (DAI) (Chassaing et al., 2014).

#### Euthanasia of Mice and Collection of Biological Material

At the end of the experimental model, mice were euthanized by inhalation of isoflurane (BioChimico, Itatiaia, RJ, Brazil) followed by cervical dislocation, as preconized by Norm no. 37 of the National Council for Control of Animal Experimentation (CONCEA). Only after complete inactivity of the animals biological material and tissues were collected. Colons of animals were removed from the ileocecal junction up to the anus, washed and measured; the material was then processed and fragmented, the distal colon being used for histological analyses and the medium/proximal colon being used for protein analyses (Geboes et al., 2000; de Paula-Silva et al., 2016).

#### Processing of Histological Sections

Distal colon fragments harvested from mice were kept overnight in 4% paraformaldehyde (Synth, Diadema, SP, Brazil) at −4°C. For processing, samples were placed in plastic cassettes and washed with decreasing polarity solutions for 1 h each for dehydrating as follows: 70% ethanol, 95% ethanol, absolute ethanol (3x) and xylol (2x) (Synth, Diadema, SP, Brazil). Afterward, samples were embedded in paraffin (Merck, Darmstadt, HE, Germany) pre-heated at 60°C. Then, 5 µm serial histological sections were obtained using a microtome (Leica Biosystems, Wetzlar, HE, Germany) and placed upon poly-lysine treated slides (Sigma-Aldrich, St. Louis, MO, United States). Prior to staining, slides were deparaffinized by pre-heating at 60°C, washed and rehydrated.

#### Histological Analyses of the Colon

Deparaffinated and hydrated slides were stained with filtered Harris hematoxylin (Sigma-Aldrich, St. Louis, MO, United States) for 2 min; slides were washed with 80% ethanol and then stained with filtered eosin for 1 min. After washing with distilled water, slides were dehydrated and mounted using a coverslip and Entelan^®^ (Sigma-Aldrich, St. Louis, MO, United States).

For histological analysis, slides were assessed with a high-power objective (×40) in a Zeiss Axio Imager 2^®^ microscope (Carl Zeiss, Oberkochen, WB, Germany) and the images obtained were processed using Zeiss Zen^®^ software (Carl Zeiss, Oberkochen, WB, Germany). Histological evaluation was performed in a qualitative manner by comparing normal colon histology of control mice with that of DSS-treated animals. Histological parameters assessed were: epithelium integrity and ulceration, loss of crypt structure, presence of edema, hydropic vacuolar degeneration, crypt dysplasia, crypt abscess and presence of inflammatory infiltrate, both in lamina propria and submucosa (Chassaing et al., 2014; de Paula-Silva et al., 2016).

#### Immunohistochemical Detection of Annexin A1 and CD68

Deparaffinated and hydrated slides were placed in citrate buffer at 96°C for 30 min. Endogenous peroxidase activity was blocked with 12% hydrogen peroxide (Synth, Diadema, SP, Brazil) for 30 min. Blocking of unspecific epitopes and tissue permeabilization was carried out by incubating the sections with tris-buffered saline (Tris-HCl buffer 200 mM, sodium chloride 1.37 M, all from Synth, Diadema, SP, Brazil) containing 10% bovine serum albumin (Sigma-Aldrich, St. Louis, MO, United States) and 0.1% Tween-20 (Synth, Diadema, SP, Brazil) (TBS-BSA). Then, two serial sections of a same organ were incubated overnight at 4 °C with either polyclonal primary anti-annexin A1 (Invitrogen, Waltham, MA, United States) or anti-CD68 (Abcam, Cambridge, CBE, United Kingdom) antibodies at dilutions of 1/250 and 1/25, respectively, diluted in TBS-BSA. Negative controls incubated only with TBS-BSA were also prepared. Then, slides were washed with TBS-BSA and incubated with anti-IgG secondary antibodies conjugated with horseradish peroxidase (HRP) (Invitrogen, Waltham, MA, United States) (Abcam, Cambridge, CBE, United Kingdom) at an 1/200 dilution for 1 h. The sections were washed with TBS-BSA and positive staining was detected using 3,3′-diaminobenzidine (DAB) (Sigma-Aldrich, St. Louis, MO, United States) for a period of 1.5 min. Then, sections were counterstained with hematoxylin (Sigma-Aldrich, St. Louis, MO, United States) and finally mounted with Entelan^®^ (Sigma-Aldrich, St. Louis, MO, United States) under a coverslip.

Immunohistochemical analysis was carried out using a Zeiss Axio Imager 2^®^ microscope (Carl Zeiss, Oberkochen, WB, Germany) and the images obtained were processed using Zeiss Zen^®^ software (Carl Zeiss, Oberkochen, WB, Germany). First at a lower power magnification (×10) slides were screened for “hot spots” areas of CD68^+^ cells, which were identified as brownish-yellow stained cells of monocyte/macrophage-like morphology. Using then higher power magnification (×100), these cells were counted in a total of twelve 0.2 mm^2^ fields and averaged. Next, in corresponding areas in adjacent serial sections the intensity of DAB signal related to AnxA1 staining was determined in an arbitrary scale ranging from 0 to 255. Image processing, counting of cells and determination of intensity of DAB staining were carried out using ImageJ^®^ software (Rueden et al., 2017).

#### Culturing of Explants and Quantification of Cytokines and MMP-9

Colonic tissue fragments obtained at the end of the experimental model were washed with sterile 0.9% saline solution so debris were removed and placed in 24-well plates in 1 ml of Dulbecco’s Modified Eagle’s Medium (DMEM) (Vitrocell Embriolife, Campinas, SP, Brazil) containing 10% of fetal bovine serum (FBS) (Vitrocell Embriolife, Campinas, SP, Brazil) and 0.1% of antibiotics (streptomycin, amphotericin and penicillin) (Vitrocell Embriolife, Campinas, SP, Brazil). Explants remained in culture at 37°C under controlled 5% CO_2_ atmosphere for a 24 h period. Next, the supernatant culture medium was collected and used for quantification of TNFα, IL-10 and metalloproteinase-9 (MMP-9) via enzyme-linked immunoassay (ELISA). Analysis was carried out using BD Opteia^®^ and DuoSet^®^ commercial kits according to manufacturer instructions (BD Biosciences, Franklin Lakes, NJ, United States) (R&D Systems, Minneapolis, MN, United States).

#### Processing of Colonic Tissue and Western Blotting Analysis

Fragments of the colonic tissue collected at the end of the conducting of the experimental model were immersed in 200 µl of radioimmunoprecipitation assay (RIPA) buffer (Tris-HCl buffer 50 mM pH 7.4, sodium deoxycholate 0.5%, dodecyl sodium sulfate 0.1%, sodium chloride 150 mM, all from Synth, Diadema, SP, Brazil) containing protease inhibitors (Sigma-Aldrich, St. Louis, MO, United States) and then processed using a tissue homogenizer T10-Basic Ultra-Turrax^®^ (IKA, Staufen, BW, Germany). The resulting suspensions were centrifuged at 5,000 g in a Sorvall ST 8R^®^ centrifuge (Thermo Fisher Scientific, Waltham, MA, United States) for 5 min for removal of non-fragmented tissue. The resulting solutions were sonicated in a Vibra-Cell VCX 500^®^ sonic bath (Sonics, Newtown, CT, United States) for complete cell lysis. The amount of protein in the homogenates was quantified via Bradford assay (Bradford, 1976). Protein homogenates were kept at −80°C until further Western Blotting tests were carried out.

Colon tissue homogenates were subjected to protein separation by sodium dodecyl sulfate polyacrylamide gel electrophoresis (SDS/PAGE) in 12% polyacrylamide gels at 100 V for 90 min; proteins were then transferred to a polyvinylidene fluoride (PVDF) membrane (Merck, Darmstadt, HE, Germany) at 400 mA for 120 min. Membranes were blocked with a solution of TBS containing 5% BSA (Sigma-Aldrich, St. Louis, MO, United States) and then incubated with either primary anti-AnxA1 antibody (Invitrogen, Waltham, MA, United States) or primary anti-PPARγ antibody (Thermo Fisher Scientific, Waltham, MA, United States), both at an 1/1,000 dilution, *overnight* at 4°C. The membranes were then washed with TBS and incubated with secondary anti-rabbit HRP conjugated antibody (Invitrogen, Waltham, MA, United States, Cat. NA9340V) at a 1/5,000 dilution for 120 min. Proteins were assessed by chemiluminescence using enhanced-chemiluminescent reagents WestPico SuperSignal^®^ (Thermo Fisher Scientific, Waltham, MA, United States), in an Amershan Imager 600^®^ (GE Healthcare, Chicago, IL, United States). Band intensities were quantified using ImageJ^®^ and are expressed as normalized optometric density units relative to ß-actin (Sigma-Aldrich, St. Louis, MO, United States) protein levels. All Western Blotting equipment used was from Bio-Rad (Bio-Rad, Hercules, CA, United States).

### *In Vitro* Experimentation

#### Cell Lines Used

RAW 264.7 cells (immortalized murine macrophages) were obtained from the Rio de Janeiro Cell Bank (BCRJ, Rio de Janeiro, RJ, Brazil) and cultured in DMEM high glucose medium (4,500 pg/ml) containing 10% FBS and 1 mM sodium pyruvate (all from Vitrocell Embriolife, Campinas, SP, Brazil). Cells were kept at 37°C under controlled CO_2_ atmosphere of 5%. Cell medium was changed every 2–3 days and cells were subcultured every 4–5 days when cells were about to become fully confluent using trypsin 0.01% containing 0.02% ethylenediamine tetra acetic acid (EDTA) (all from Vitrocell Embriolife, Campinas, SP, Brazil).

#### Knockdown of AnxA1 Expression in Macrophages

Knockdown of AnxA1 on RAW 264.7 cells was carried out using commercial Mission^®^ pLKO.1 plasmids (#Addgene 10,878) containing four different short-hairpin RNA (shRNA) sequences designed to be complementary to the 3′-UTR murine AnxA1 mRNA sequence (Sigma-Aldrich, St. Louis, MO, United States). Transformed *E. coli* purchased in glycerol stocks were grown according to manufacturer instructions (Sigma-Aldrich, St. Louis, MO, United States).

Plasmids were purified from culture medium containing transformed bacteria using the commercial kit PureLink^®^ HiPure Plasmid Maxiprep (Thermo Fisher Scientific, Waltham, MA, United States), according to manufacturer instructions. After purification, plasmid concentration was assessed using a Nanodrop 2000^®^ spectrophotometer (Thermo Fisher Scientific, Waltham, MA, United States).

HEK293FT cells (renal embryo cells, American Type Culture Collection, Manassas, VA, United States) were plated on 10 cm dishes at a density of 2 x 10^6^ cells per plate and cultured with DMEM containing 10% FBS, 1 mM sodium pyruvate and 1 mM glutamine (Vitrocell Embriolife, Campinas, SP, Brasil) at 37°C under controlled CO_2_ atmosphere of 5%. Once cells reached 80% confluence, they were washed and culture medium was renewed with medium containing 25 µM chloroquine (Sigma-Aldrich, St. Louis, MO, United States), which remained on the cells for 30 min. After this period, these cells were transfected by calcium phosphate precipitation. For such, 3 µg of each accessory plasmid (pRSV rev, pMDLg/pRRE and pHCMV-G), 0.5 µg of fluorescence reporter plasmid (pEGFP) and 8 µg of the control plasmids (pLKO.1 empty vector and a plasmid containing scrambled, non-functional sequence, used for mock-transfection) or shRNA plasmids (Mission^®^ pLKO.1 AnxA1 shRNA plasmid, Sigma-Aldrich, St. Louis, MO, United States) were added to 500 µl of a 0.25 M CaCl_2_ solution. After that, 500 µl of HEPES Buffer Saline 2X (280 mM NaCl, 1.5 mM Na_2_HPO_4_ and 50 mM HEPES; pH 7.0, all from Synth, Diadema, SP, Brazil) were added to the transfection mixture while vortexing and then allowed to rest for eration. HEK293FT cells were incubated for 6 h in culture medium containing 25 µM of chloroquine and the transfection mixture. At the end of this period, culture medium was renewed and the cells remained in incubator at 37°C under 5% CO_2_ for further 42 h. Forty-eight hours after transfection, the supernatants containing lentiviral particles were harvested, centrifuged at (3,000 rpm for 15 min) and filtered using 0.45 µm filters. All procedures involving manipulation of lentiviral material were carried out following special safety regulations as preconized by Brazilian health and safety laws.

RAW 264.7 cells were previously plated in 6-well plates at a density of 2 x 10^5^ cells per well for lentiviral transduction and cultured as described in item 2.2.1. These cells were incubated with a solution of renewed media, containing 1:3 (2 ml per well) diluted virus and supplemented with 10 μg/ml of polybrene (EMD Millipore, Billerica, MA, United States). RAW 264.7 cells were kept in incubation for 24 h at 37°C under controlled CO_2_ atmosphere. Puromycin (Santa Cruz Biotechnology, Dallas, TX, United States) was added to the culture medium at a final concentration of 7 μg/ml for 48 h for selection of stable transduced cells.

After puromycin selection, a total of 5 x 10^5^ cells were lyzed in 50 µl of RIPA buffer containing protease inhibitors; Western Blotting procedure for validation of AnxA1 knockdown was identical to the procedure described in item 2.1.9. Out of the four shRNA sequences tested, the one which led to most efficient AnxA1 knockdown was selected to be used for all further experiments. The growth of mock-transfected and AnxA1-knockdown cells was assessed daily by manual counting for 7 days and compared to the growth of wild type RAW 264.7 cells.

Information regarding shRNA sequences used, AnxA1 knockdown efficiency and cell growth rates are detailed in Supplementary Material (Supplementary Figure S2).

#### Cell Treatments

RAW 264.7 cells were cultured as described in item 2.2.1. For experimentation, cells were cultured in 24-well plates at a density of 2 x 10^5^ cells/well (for flow cytometry and ELISA tests) or in 12-well plates at a density of 5 x 10^5^ cells/well (for Western Blotting tests). After cells had adhered, the culture medium was replaced with fresh medium and cells were treated.

Cells were treated with pioglitazone, a specific PPARγ ligand, at a concentration of 10 µM (Sigma-Aldrich, St. Louis, MO, United States) for 24 h. *E. coli* lipopolysaccharide 026.B6 at a concentration of 1 μg/ml was added to the cells 1 h after pioglitazone was added. For specific experiments, cells were also treated with GW9662, a PPARγ antagonist, at a concentration of 10 µM (Tocris, Bristol, BRS, United Kingdom) for 30 min prior to treatment with pioglitazone. Control cells were treated only with the vehicle used for solubilization of pioglitazone, 0.1% dimethyl sulfoxide (DMSO) (Synth, Diadema, SP, Brazil).

At the end of each treatment, supernatants from the culture medium were collected and stored at −80°C until further ELISA tests and cells were washed with phosphate buffer saline (PBS) and harvested with trypsin 0.01% containing 0.02% EDTA (all from Vitrocell Embriolife, Campinas, SP, Brazil) for either flow cytometry or Western Blotting tests.

Details of the experimental protocol for cell treatment are graphically described in Supplementary Material (Supplementary Figure S3).

#### Assessment of Cytokine and MMP-9 Secretion

After treatments, cell supernatants were collected for assessment of cytokine and MMP-9 secretion via ELISA. Quantification was carried out using commercial ELISA kits following manufacturer instructions (BD Biosciences, Franklin Lakes, NJ, United States). TNFα and IL-10 cytokines were assessed.

#### Assessment of ROS Production and Expression of Surface Markers

After treatments, cells were collected and production of reactive oxygen species (ROS) and expression of adhesion molecules and of formyl peptide receptors 1 and 2 (FPR1 and FPR2) on cell surface were assessed via flow cytometry. For assessment of reactive oxygen species (ROS) production, 5x10^4^ cells were separated from the total amount of treated cells, washed with PBS and incubated with 0.3 mM dichlorofluorescein diacetate (DCFH-DA) (Sigma-Aldrich, St. Louis, MO, United States) solution diluted in PBS. Cells were kept at 37°C under controlled CO_2_ atmosphere of 5% for 30 min. After this incubation period, cold PBS was added to the cells. Lastly, cells were taken to an Accuri C6^®^ flow cytometer (BD Biosciences, Franklin Lakes, NJ, United States) and a minimum of 10,000 events were acquired per sample. For assessment of cell surface adhesion molecules, 2 x 10^5^ cells were plated, treated, collected, washed with PBS and incubated with phycoerythrin-labeled (PE) anti-CD54 (BD Biosciences, Franklin Lakes, NJ, United States, Cat. 553263) or PE-labeled anti-CD62L (BD Biosciences, Franklin Lakes, NJ, United States, Cat. 553151), both at a 1/100 dilution in PBS, for 30 min. The protocol was the same for assessment of FPR1 and FPR2, but cells were incubated with either fluorescein isothiocyanate (FITC) labeled anti-FPR2 antibody (Bioss, Woburn, MA, United States) for 30 min or primary anti-FPR1 antibody (CliniSciences, Nanterre, IDF, France) for 2 h, both at a 1/100 dilution; cells previously incubated with anti-FPR1 antibody were washed with PBS and incubated with PE-labeled anti-IgG secondary antibody (Abcam, Cambridge, CBE, United Kingdom) at a 1/200 dilution for 30 min. Lastly, cells were again washed and taken to an Accuri C6^®^ flow cytometer (BD Biosciences, Franklin Lakes, NJ, United States) and a minimum of 10,000 events were acquired per sample.

#### Assessment of AnxA1 Expression and Cleaving and PPARγ Expression

For assessment of AnxA1 cleaving, cells were harvested after treatments as described in item 2.2.3 and cell lysates were prepared using RIPA buffer containing protease inhibitors; quantification of protein content via Bradford assay and Western Blotting analyses of quantification of AnxA1 and of assessment of AnxA1 cleaving were carried out as described in item 2.1.9.

For assessment of AnxA1 and PPARγ expression, cells were harvested, fixed with 4% paraformaldehyde for 30 min (Synth, Diadema, SP, Brazil), washed with 0.1 M glycin (Synth, Diadema, SP, Brazil) and permeabilized with 0.01% Triton-X for 30 min (Sigma-Aldrich, St. Louis, MO, United States). Cells were washed with PBS between all steps. Then, cells were incubated overnight with either primary anti-AnxA1 (BD Biosciences, Franklin Lakes, NJ, United States) or anti-PPARγ (Thermo Fisher Scientific, Waltham, MA, United States) antibodies in a 0.1% BSA and 0.01% sodium azide solution (Thermo Fisher Scientific, Waltham, MA, United States) at an 1/100 dilution for either. Cells were then washed and incubated with PE conjugated secondary anti-IgG antibody at a 1/200 dilution for 30 min. Cells were then taken to an Accuri C6^®^ flow cytometer (BD Biosciences, Franklin Lakes, NJ, United States) and a minimum of 10,000 events were acquired.

#### Assessment of ERK Phosphorylation

Phosphorylation of extracellular signal-regulated kinase (ERK) proteins was carried out via flow cytometry. Mock-transfected and AnxA1-knockdown RAW 264.7 cells were harvested after treatments and processed the same manner as described in item 2.2.3. Cells were incubated overnight with primary anti-ERK1/2 and anti-pERK1(Thr202)/2(Thr185) antibodies (BD Biosciences, Franklin Lakes, NJ, United States) at 1/100 and 1/200 dilutions, respectively. Cells were then washed and incubated with FITC and PE conjugated secondary anti-IgG antibodies at dilutions 1/200 for 30 min. Finally, cells were taken to an Accuri C6^®^ flow cytometer (BD Biosciences, Franklin Lakes, NJ, United States) and a minimum of 10,000 events were acquired.

### Statistical Analysis

Data were analyzed using GraphPad Prism seven software (Graphpad Software^®^, San Diego, CA, United States). For analysis of clinical parameters two factor analysis of variance tests (Two-Way ANOVA) followed by Bonferroni post-hoc tests were performed. For comparison between number of stained macrophages and intensity of AnxA1 signal in DAB-stained tissue sections, linear regressions followed by Pearson's correlation tests were performed. For all other experiments performed, data were analyzed using when appropriate either Student's t tests or one factor analysis of variance (One-Way ANOVA) followed by Tukey post-hoc tests in order to assess differences between the evaluated groups. Differences between assessed means and correlation coefficients were considered statistically significant assuming *p* < 0.05. All results are shown as mean ± standard-error of the mean.

## Results

### Pioglitazone Treatment Attenuates Dextran Sodium Sulfate-Induced Colitis Progression of Wild Type, but Not AnxA1^−/−^ Mice

**FIGURE 1:**
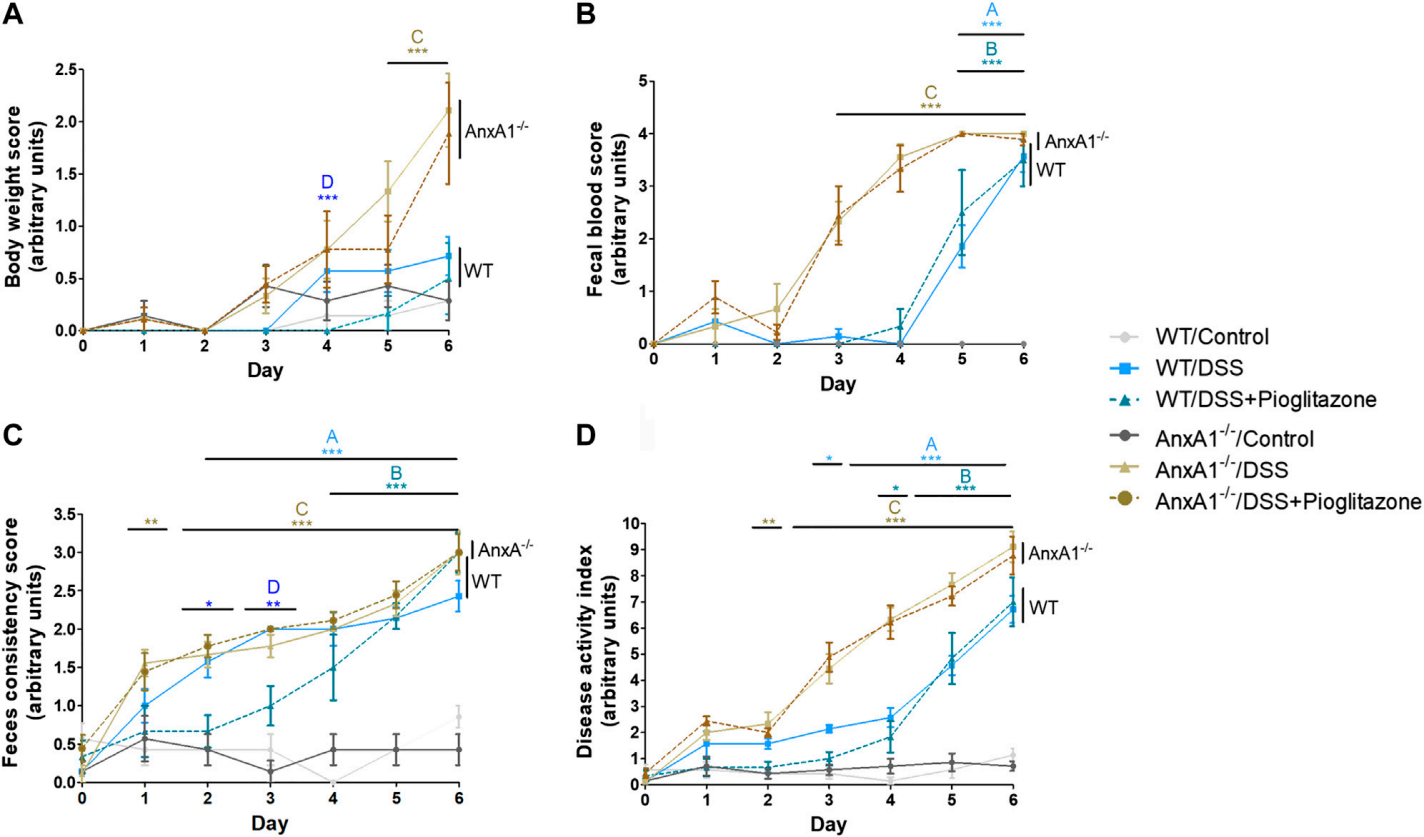
Clinical parameters throughout progression of experimental colitis. Experimental colitis was induced in wild type or AnxA1^−/−^ mice via ingestion of DSS and mice were treated with pioglitazone daily at a 10 mg/kg dose for a 6 days period. Throughout the development of the experimental model, loss of body weight **(A)**, rectal bleeding **(B)** and feces consistency **(C)** were assessed daily, resulting in the disease Activity Index **(D)**. *, **, ***p < 0.05, 0.01, 0.001 in comparison to another group as specified by the letters above each curve: A: WT/Control x WT/DSS; B: WT/Control x WT/DSS + Pioglitazone; C: AnxA1^−/−^/Control x AnxA1^−/−^/DSS and/or AnxA1^−/−^/DSS + Pioglitazone; D: WT/DSS x WT/DSS + Pioglitazone. Data were statistically analyzed using Two-Way ANOVA followed by Bonferroni post-test (*n* = 6). Results are expressed as mean ± SE.

Clinical manifestations of DSS-induced colitis were assessed as body weight loss, rectal bleeding and consistency of feces, their sum resulting in a greater DAI in DSS-treated than control mice. All these disease parameters were even more pronounced in AnxA1^−/−^ mice, confirming AnxA1^−/−^ mice are more susceptible to damage caused by DSS (Babbin et al., 2008; Leoni et al., 2015; de Paula-Silva et al., 2016). The intraperitoneal administration of pioglitazone reduced body weight loss and increased feces consistency between days 2 and 4 of the disease in WT mice. Pioglitazone treatment did not attenuate clinical parameters of colitis in AnxA1^−/−^ mice, and disease scores remained comparable to that of non-treated animals (Figures 1A–D). These data evidence pioglitazone attenuates the development of DSS-induced colitis in an AnxA1 dependent manner.

Also, in order to verify whether these beneficial clinical effects caused by pioglitazone treatment could be influenced or not by AnxA1 knockdown or by DSS intake due to changes in PPARγ expression, PPARγ expression was assessed in tissue homogenates from colon fragments. PPARγ expression in AnxA1^−/−^ mice was found to be at the same levels of WT mice, regardless of any treatments, thus showing any effects on clinical parameters (and on all other further effects described *in vivo*) caused by pioglitazone treatment in mice were likely not influenced by changes in PPARγ expression (Supplementary Figure S4).

### Pioglitazone Treatment Preserves Colon Extension and Histoarchitecture of Wild Type, but Not AnxA1^−/−^ Mice

**FIGURE 2:**
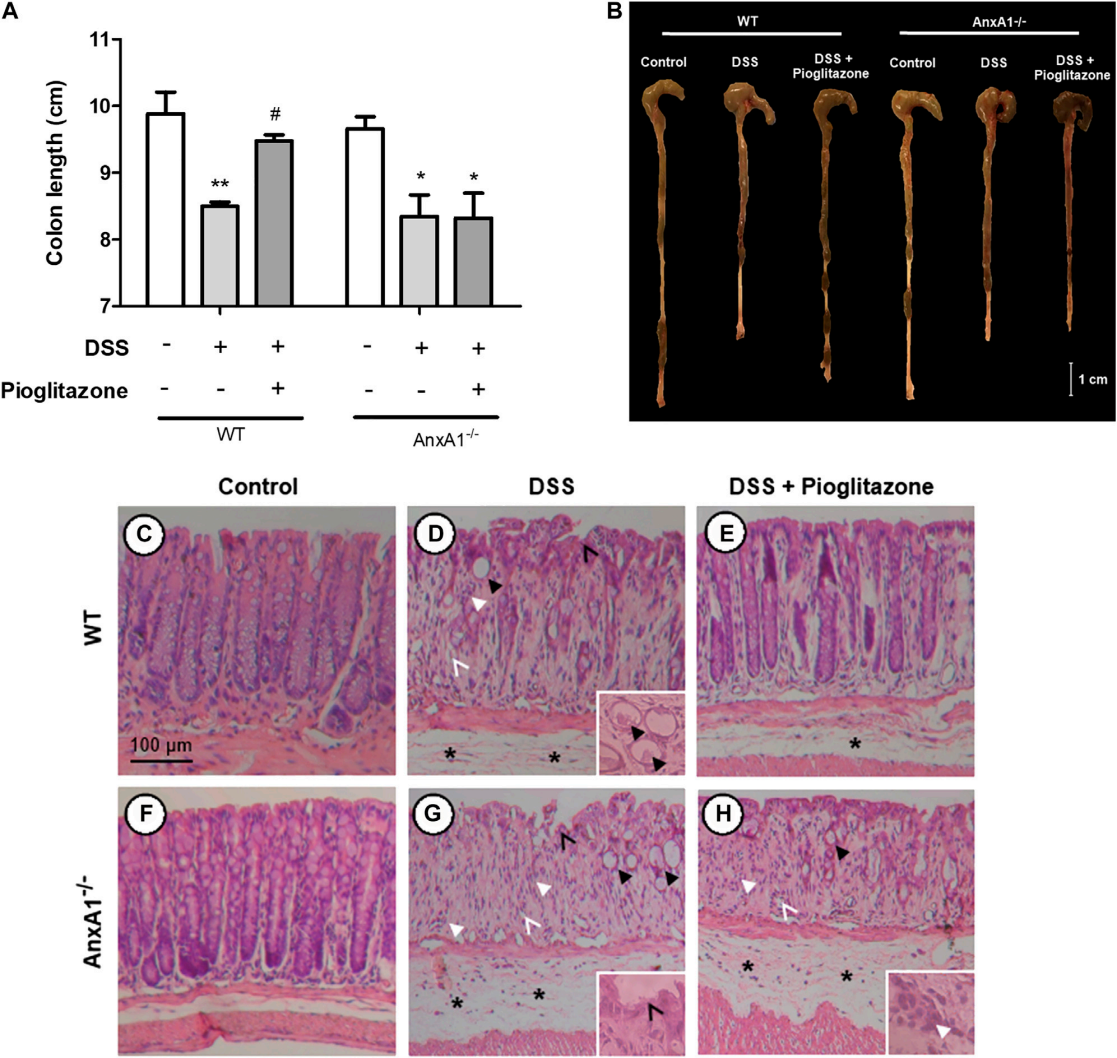
Large intestine extension and colon histoarchitecture on tissue sections. Wild type and AnxA1^−/−^ mice were euthanized at the end of the experimental model; large intestines were harvested and their extensions measured **(A,B)**. Paraffin-embedded sections were prepared from distal colon fragments and HE-stained; tissue histoarchitecture and presence of DSS lesions were evaluated via optical microscopy for both wild type **(C–E)** and AnxA^−/-^ mice **(F–H)** (*n* = 6). Asterisks in histological images indicate edema; thicker black arrows indicate dysplastic crypts (crypt abscess in the detail in **D**); thinner black arrows indicate epithelium ulceration; thicker white arrows indicate leukocyte infiltration; thinner white arrows indicate hydropic vacuolar degeneration. *, **p < 0.05, 0.01 in comparison to respective control group; ^#^ *p* < 0.05 in comparison to respective DSS-treated group. Data were statistically analyzed using One-Way ANOVA followed by Tukey’s post-test (*n* = 6). Results are expressed as mean ± SE.

The extension of large intestines of DSS-treated mice was significantly reduced in comparison to samples of control mice. Pioglitazone treatment attenuated such reduction only in WT mice, as intestines harvested from DSS treated AnxA1^−/−^ mice treated with pioglitazone had the same extension as AnxA1^−/−^ mice which received DSS only (Figures 2A,B). These data corroborate the findings related to assessment of clinical parameters confirming pioglitazone treatment halts DSS-induced colitis progression in WT but not in AnxA1^−/−^ mice.

Histopathological analyses of intestines showed administration of DSS induced tissue injuries not observed in control mice, which resemble those found in human IBD (Perse and Cerar, 2012) (Figures 2C–E). DSS administration caused loss of tissue structure alongside ulcerations, loss of crypt structure, presence of edema and of inflammatory infiltrates, crypt abscesses, crypt dysplasia and vacuolar hydropic degeneration. Such effects were more pronounced in AnxA1^−/−^ mice (Figures 2F–H). Pioglitazone treatment attenuated loss of tissue morphology in WT mice, as there was less edema and crypts were still preserved to some extension (Figure 2E). Beneficial effects of pioglitazone treatment were not observed in tissue sections of DSS-treated AnxA1^−/−^ mice (Figure 2H). In association with clinical parameters, these findings show pioglitazone preserves colon histoarchitecture and reduces damage caused by DSS administration, the presence of AnxA1 being an important role player for such effects.

### Inhibitory Effects of Pioglitazone on Secretion of Inflammatory Cytokines by Colon Tissue Is Modulated by AnxA1

**FIGURE 3:**
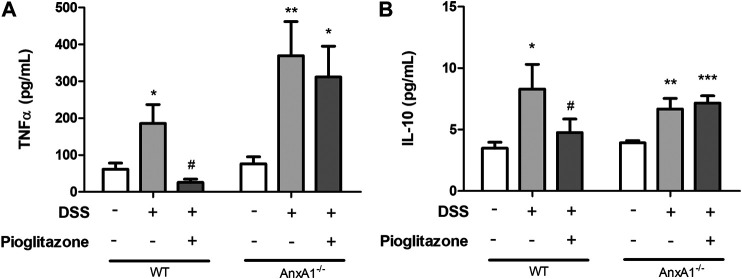
Cytokines from tissue explants. Wild type and AnxA1^−/−^ mice were euthanized at the end of the experimental model; large intestines were harvested and a fragment of the colon was cultured; after 24 h, supernatants were collected and secretion of TNFα **(A)** and IL-10 **(B)** was assessed via ELISA. *, **, ***p < 0.05, 0.01, 0.001 in comparison to the control group; ^#^ *p* < 0.05 in comparison to the DSS-treated group. Data were statistically analyzed using One-Way ANOVA followed by Tukey’s post-test (*n* = 6). Results are expressed as mean ± SE.

Analysis of inflammatory cytokines evidenced increased secretion of TNFα and IL-10 from WT or AnxA1^−/−^ inflamed colon explants. Treatment with pioglitazone reduced the secretion of both cytokines from colon explants of WT mice, but not from colon explants of AnxA1^−/−^ mice, suggesting pioglitazone relies on expression of AnxA1 to attenuate the secretion of inflammatory cytokines (Figures 3A–D). These data, altogether with those describing assessment of clinical parameters, intestine extension and colon histoarchitecture, evidence pioglitazone treatment modulates the development of DSS induced colitis in an AnxA1 dependent manner.

### Pioglitazone Prevents Cleaving of AnxA1 in Colon Tissue

**FIGURE 4:**
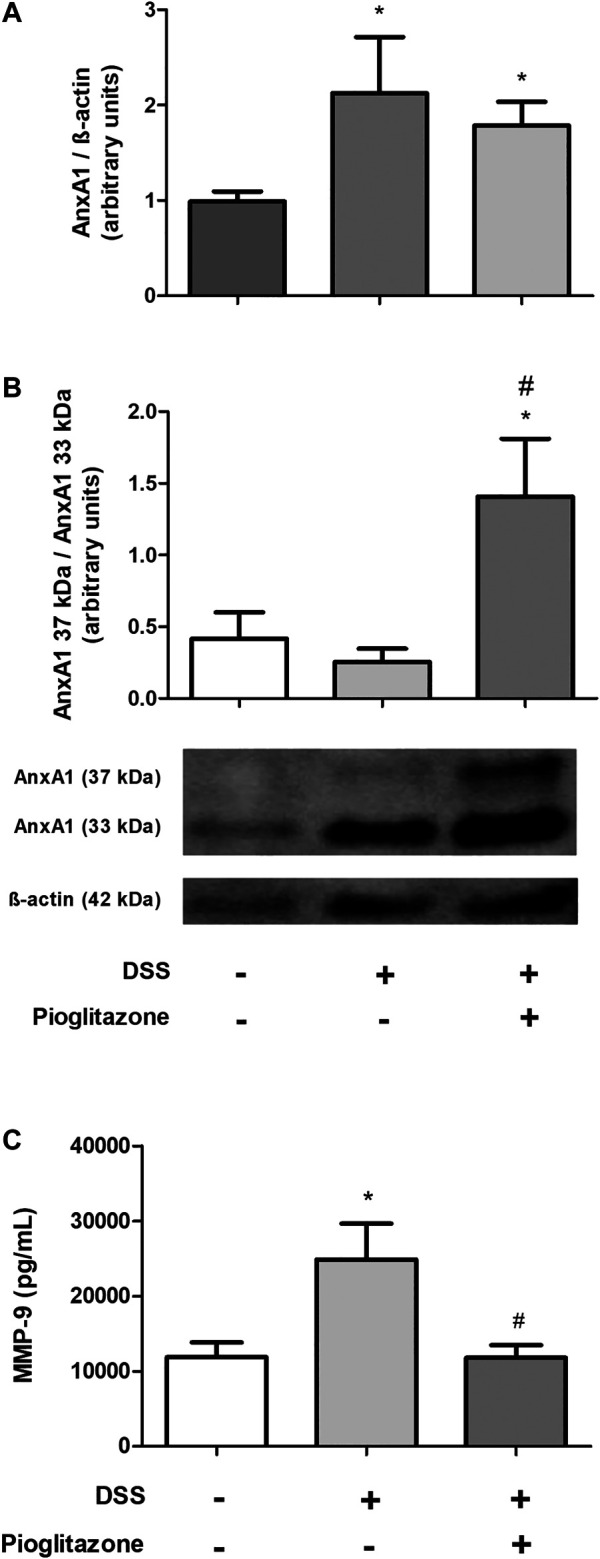
Cleaving of AnxA1 in colon tissue. Wild type and AnxA1^−/−^ mice were euthanized at the end of the experimental model; large intestines were harvested and protein homogenates were prepared; expression and cleaving of AnxA1 were assessed via *Western Blotting* **(A,B)** and MMP-9 expression was assessed via ELISA **(C)**. *p < 0.05 in comparison to the control group; ^#^ *p* < 0.05 in comparison to the DSS-treated group. Data were statistically analyzed using One-Way ANOVA followed by Tukey’s post-test (*n* = 6). Results are expressed as mean ± SE.

As it was evidenced AnxA1 is necessary for the anti-inflammatory effects of pioglitazone on DSS-induced colitis, its expression and cleaving status were assessed in WT mice. DSS administration increased expression of AnxA1 in the inflamed colon, regardless of treatment with pioglitazone (Figure 4A; Supplementary Figures S5A–D). However, pioglitazone treatment prevented AnxA1 cleaving into non-functional 33 kDa fragments, preserving whole the functional 37 kDa protein (Figure 4B; Supplementary Figures S5A–D). Also, while DSS-treatment increased the expression of MMP-9 as assessed in colon tissue homogenates, treatment with pioglitazone decreased such expression (Figure 4C). These data demonstrate pioglitazone treatment preserves the structure of full length 37 kDa AnxA1 and decreases the expression of MMP-9 induced by DSS, which was previously shown to be involved with AnxA1 cleaving in mice intestinal epithelial cells (Leoni et al., 2015).

### Dextran Sodium Sulfate-Induced Colitis Promotes Recruitment of AnxA1-Releasing Macrophages Regardless of Pioglitazone Treatment

**FIGURE 5:**
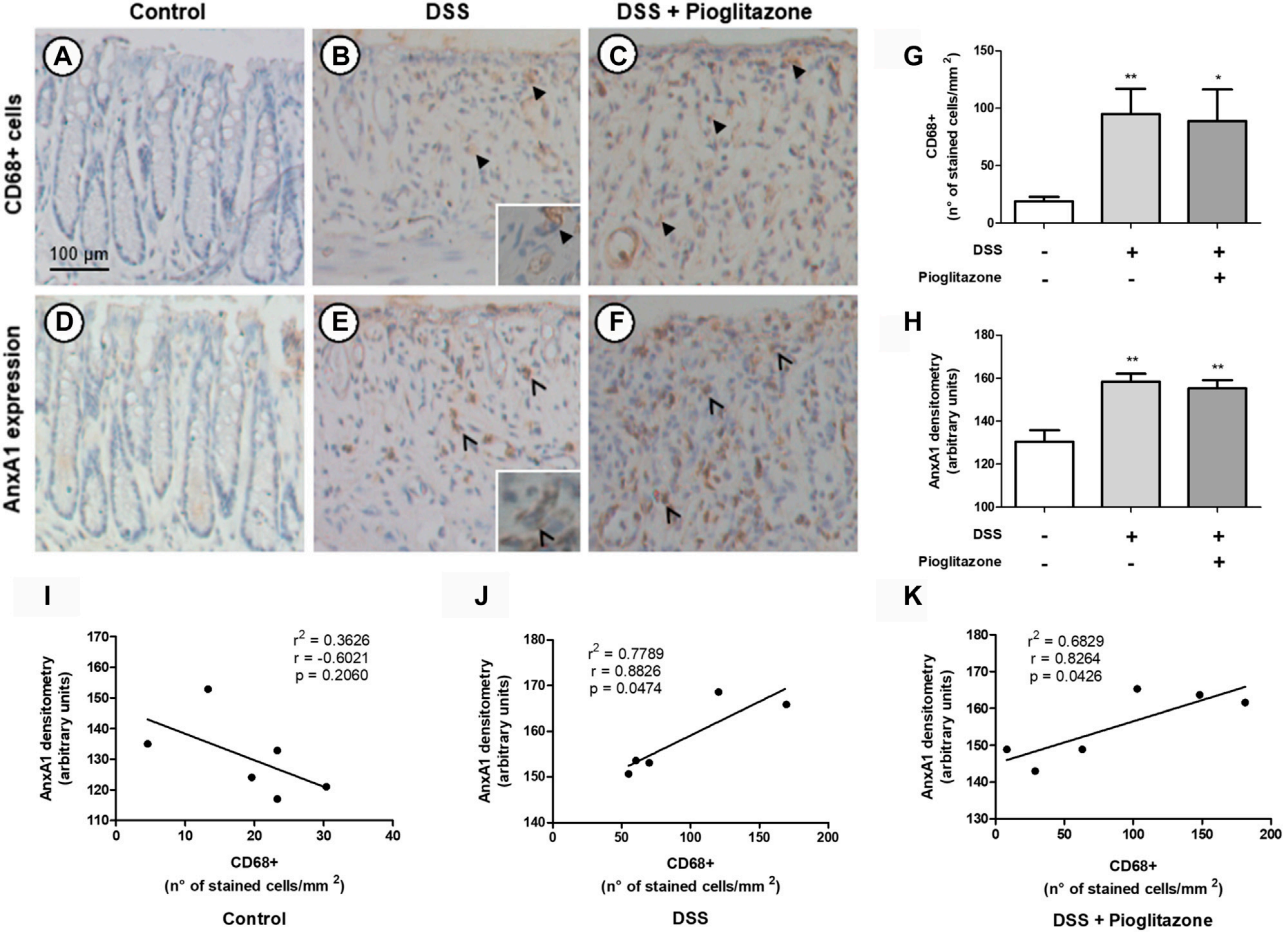
Expression of AnxA1 and presence of CD68^+^ macrophages in tissue sections. Wild type and AnxA1−/− mice were euthanized at the end of the experimental model; large intestines were harvested and colon paraffin-embedded sections were prepared. Number of CD68^+^ macrophages and staining intensity corresponding to AnxA1 were assessed via DAB-staining in serial sections **(A–F)** (thicker black arrows indicate CD68^+^ cells; thinner black arrows indicate staining corresponding to AnxA1 in macrophage-like cells), quantified **(G,H)** and correlated **(I–K)**. *, **p < 0.05, 0.01 in comparison to the control group. Data were statistically analyzed using One-Way ANOVA followed by Tukey’s post-test (*n* = 6). Results are expressed as mean ± SE. Corresponding areas in each serial section were analyzed using Pearson’s correlation test.

Analysis of colon sections showed higher number of CD68 ^+^ macrophages in the colon of DSS-treated mice in comparison to control mice, regardless of treatment with pioglitazone (Figures 5A–C,G). Corresponding areas in serial sections assessed for AnxA1 staining evidenced a greater presence of this protein in tissue sections from DSS-treated mice in comparison to tissue sections from control mice; such increased staining corresponding to AnxA1 also was not influenced by pioglitazone treatment (Figures 5D–F,H). Correlation analysis associated AnxA1 expression with increased number of CD68 ^+^ macrophages (Figures 5I–K). These findings allow us to suggest DSS colitis leads to recruitment of AnxA1-releasing CD68 ^+^ macrophages and also increases AnxA1 secretion, and such events are unaffected by pioglitazone treatment.

### Lipopolysaccharide-Induced Inflammatory Parameters Are Attenuated by Pioglitazone in RAW 264.7 Macrophages Relying on AnxA1 Expression

**FIGURE 6:**
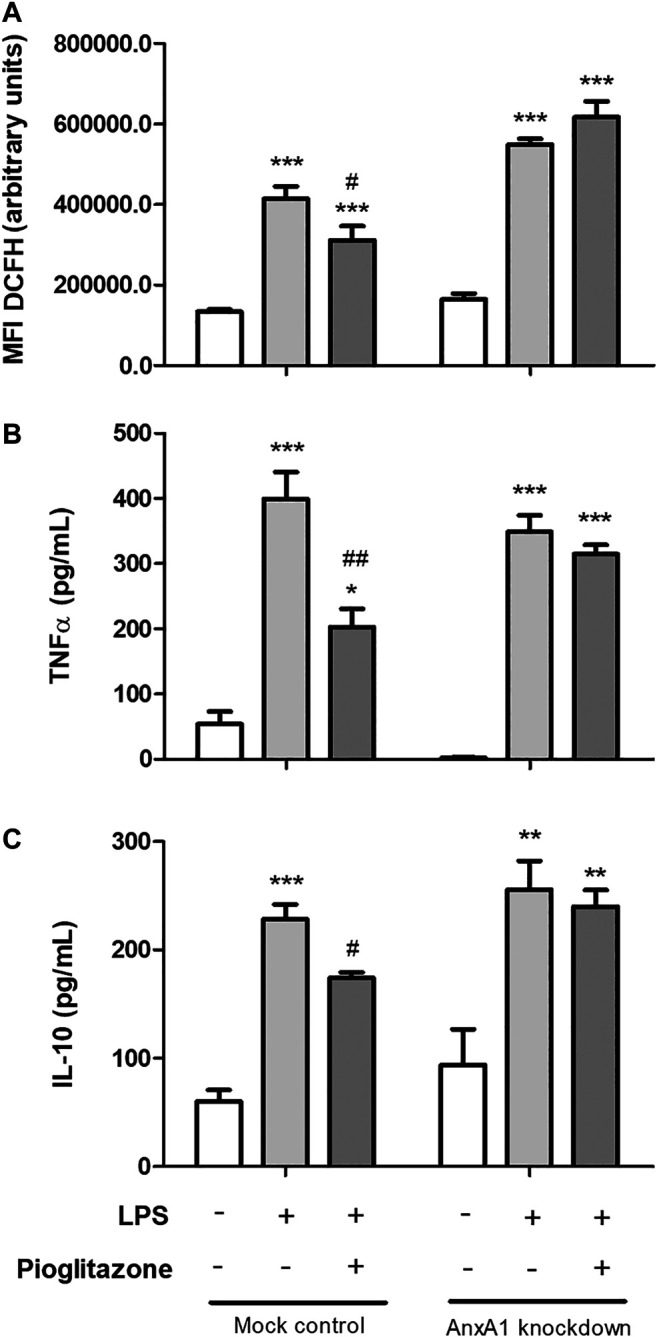
Secretion of inflammatory cytokines and ROS production by transfected RAW 264.7 macrophages. Mock-transfected and AnxA1-knockdown RAW 264.7 cells were plated and treated with 10 µM pioglitazone for 1 h followed by 1 μg/ml LPS for 24 h. Cells were harvested and ROS production was assessed via DFCH-DA via flow cytometry **(A)**; supernatants were collected and secretion of TNFα **(B)** and IL-10 **(C)** was assessed via ELISA. *, **, ***p < 0.05, 0.01, 0.001 in comparison to the control group; ^#^, ^##^ *p* < 0.05, 0.01 in comparison to the LPS-treated group. Data were statistically analyzed using One-Way ANOVA followed by Tukey’s post-test (*n* = 3). Results are expressed as mean ± SE.

The generation of AnxA1 knockdown RAW 264.7 cells and further stimulation with LPS was the strategy employed to investigate the role of AnxA1 on pioglitazone-mediated anti-inflammatory effects and the actions of pioglitazone on cleaving of AnxA1 in macrophages. LPS treatment increased the generation of ROS and secretion of TNFα and IL-10 by RAW 264.7 macrophages. These parameters were attenuated in mock-transfected cells treated with pioglitazone, but not in AnxA1-knockdown transfected cells (Figures 6A–C). LPS treatment also increased surface expression of CD54 and CD62L, which was reversed by treatment with pioglitazone in mock-transfected cells but not in AnxA1-knockdown cells (Supplementary Figures S6A,B). Thus, these data evidence AnxA1 is key for the anti-inflammatory effects of pioglitazone on macrophages, corroborating our *in vivo* data in an isolated *in vitro* system.

Following a similar rationale as investigated in mouse tissue, PPARγ expression was verified as to assess whether changes on it would influence anti-inflammatory outcomes exerted by pioglitazone treatment. Unlike the results seen in mouse tissue, PPARγ expression increased due to LPS treatment, regardless of actions of pioglitazone, but only in mock-transfected RAW 264.7 cells (Supplementary Figure S7A). Further analysis under non-inflammatory conditions revealed pioglitazone induced PPARγ expression as expected in mock-transfected cells but not in AnxA1-knockdown cells (Supplementary Figure S7B). These findings evidence that functional expression of AnxA1 is required for pioglitazone to exert its actions and induce PPARγ expression.

### Pioglitazone Prevents the Cleaving of AnxA1 in RAW 264.7 Macrophages

**FIGURE 7:**
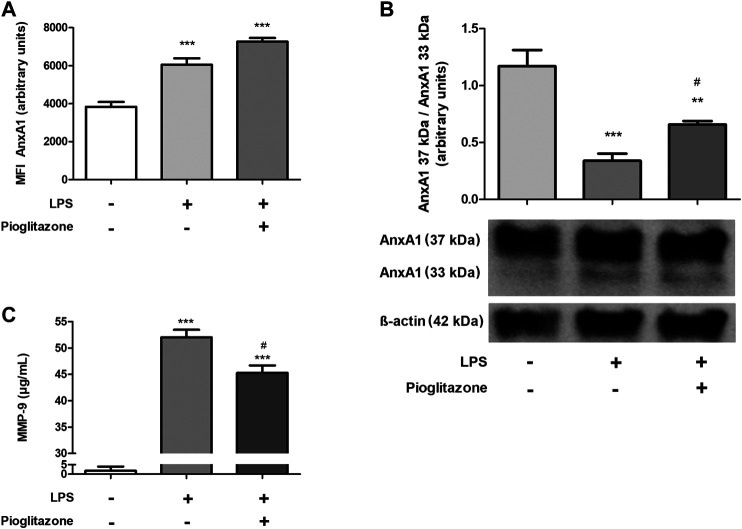
Expression and cleaving of AnxA1 in RAW 264.7 macrophages. Non-transfected (wild-type) RAW 264.7 cells were plated and treated with 10 µM pioglitazone for 1 h followed by 1 μg/ml LPS for 24 h. Cells were harvested and AnxA1 expression via flow cytometry was assessed on part of them **(A)**; protein lysates were prepared from the remaining cells and AnxA1 cleaving was assessed via Western Blotting **(B)**. Culture supernatants were collected and MMP-9 secretion was assessed via ELISA **(C)**. *, ***p < 0.05, 0.001 in comparison to the control group; ^#^ *p* < 0.05 in comparison to the LPS-treated group. Data were statistically analyzed using One-Way ANOVA followed by Tukey’s post-test (*n* = 5). Results are expressed as mean ± SE.

In the same vein as seen *in vivo*, inflammation caused by LPS increased AnxA1 expression in wild type RAW 264.7 cells, effect not reversed by treatment with pioglitazone (Figure 7A). LPS also caused AnxA1 cleaving into 33 kDa fragments, while pioglitazone prevented such cleaving (Figure 7B; Supplementary Figures S8A–J). LPS treatment also increased MMP-9 expression, and pioglitazone treatment did prevent such increase (Figure 7C). Thus, corroborating our *in vivo* data, pioglitazone is unable to control AnxA1 expression in inflammatory conditions, but can modulate its functionality by preventing its cleaving.

### Pioglitazone Actions Are PPARγ-Dependent in RAW 264.7 Macrophages

As it was determined PPARγ expression increased due to treatment with pioglitazone in mock-transfected cells, we investigated whether anti-inflammatory effects seen so far in AnxA1-expressing RAW 264.7 cells would be abrogated due to blocking of PPARγ. Increased levels of TNFα and MMP-9 induced by LPS were attenuated by treatment with pioglitazone, but not when cells have been previously treated with GW9662 (Supplementary Figures S9A,B). This evidences the anti-inflammatory actions of pioglitazone, at least those here assessed, are likely dependent on PPARγ activation.

### AnxA1 Is Required for ERK Phosphorylation Induced by Pioglitazone in RAW 264.7 Macrophages

**FIGURE 8:**
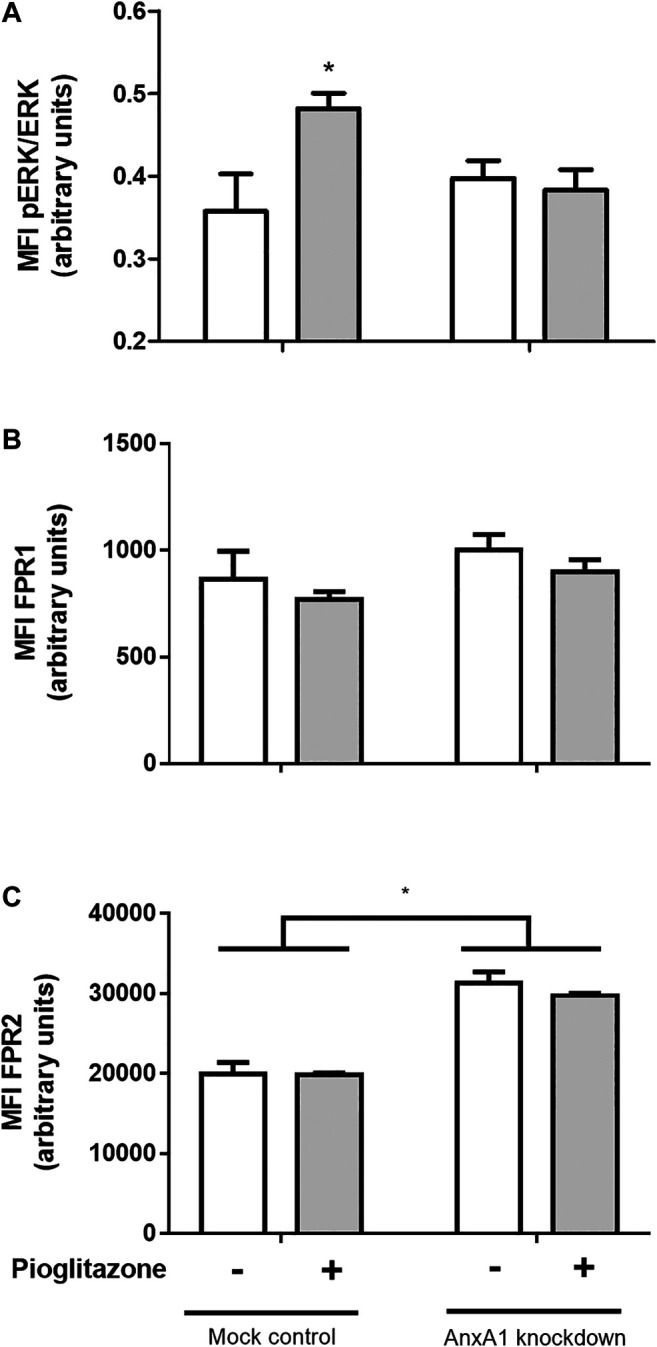
Phosphorylation of ERK and expression of FPR1 and FPR2 in transfected RAW 264.7 macrophages. Mock-transfected and AnxA1-knockdown RAW 264.7 cells were plated and treated with 10 µM pioglitazone for 1 h. Cells were harvested and both ERK phosphorylation **(A)** and expression of FPR1 **(B)** and FPR2 **(C)** were assessed via flow cytometry. *p < 0.05 in comparison to the control group. Data were statistically analyzed using One-Way ANOVA followed by Tukey’s post-test (*n* = 4). Results are expressed as mean ± SE.

ERK phosphorylation is a *downstream* pathway event following AnxA1 binding to its receptors and pioglitazone can induce ERK phosphorylation. Hence, we investigated ERK phosphorylation as a possible factor connecting the anti-inflammatory actions of pioglitazone and AnxA1. Indeed, ERK phosphorylation was increased in mock-transfected RAW 264.7 cells treated with pioglitazone, but not in AnxA1-knockdown cells, meaning *downstream* signaling after PPARγ activation requires expression of functional AnxA1 (Figure 8A). Expressions of FPR1 and FPR2, G-protein coupled receptors known to bind to AnxA1 and exert downstream signaling, of which FPR2 is the better described for anti-inflammatory effects of AnxA1, did not change due to treatment with pioglitazone in either mock-transfected or AnxA1-knockdown cells, but FPR2 expression was overall higher in AnxA1-knockdown cells, likely as a cell response to low levels of AnxA1 (Figures 8B,C). Thus, while pioglitazone does not necessarily modulate FPR2 expression, the lack of AnxA1, which is needed for ERK activation via FPR2, induces its expression.

## Discussion

Research on IBD therapy has advanced on the past few years, and inflammatory cytokines or specific cellular pathways such as JAK/STAT and phosphodiesterase-4 have all been explored as potential targets for IBD treatment (Pekow, 2017). Still, it is unlikely that a significant number of such newly-developed drugs aimed at interacting with the aforementioned targets will reach clinical practice, as several of these compounds do not display satisfactory remission rates or lead to severe adverse effects, such as increased risk for infection, malignancies or immunological disorders in larger clinical trials (Pekow, 2017; Click and Regueiro, 2019).

Thus, the search for drugs for treatment of IBDs has taken scientists to explore molecules beyond those designed to specifically treat IBDs and reassess drugs originally developed to treat other maladies (Lloyd et al., 2019; Kostoff et al., 2020). Such is the case of thiazolidinediones, PPARγ ligands originally developed for treatments of diabetes, which have been explored to some success for treatment of IBDs. While clinical trials on PPARγ ligands seem somewhat promising, obtaining of conclusive results which could take research steps further on drug repurposing has been elusive so far (Lewis et al., 2008; Liang and Ouyang, 2008; Abedi et al., 2015).

While mechanisms unveiling how thiazolidinediones ameliorate IBD progression are yet to be completely understood, data on AnxA1 roles on IBD suggest it might be a promising player on the actions of PPARγ ligands leading to anti-inflammatory effects. Indeed, our research group has evidenced AnxA1 is vital for infliximab actions on murine IBD models (de Paula-Silva et al., 2016) and that AnxA1 is a promising biomarker for assessing responsiveness to infliximab in human IBD patients (unpublished observations). We have also shown both exogenous and endogenous AnxA1 are required for pioglitazone to induce BV2 microglia cells to phagocytize apoptotic cells, and that AnxA1 can modulate PPARγ expression in such cells (da Rocha et al., 2019). Data here obtained confirm the connection between pioglitazone and AnxA1 on treatment of experimental colitis, evidencing the inhibition of AnxA1 cleaving into its inactive form as a mechanism of action of pioglitazone; therefore, we suggest AnxA1 is as a key player required for the anti-inflammatory actions of pioglitazone and thus possibly other PPARγ ligands.

Data here obtained corroborated other studies which already evidenced the beneficial actions of pioglitazone in murine models of colitis (Saubermann et al., 2002; Takagi et al., 2002; Shah et al., 2007; Huang et al., 2020) and evidenced the lack of pioglitazone effects on AnxA1^−/−^ mice demonstrating the pivotal role of AnxA1 for pioglitazone anti-inflammatory actions. However, these data do not rule out the effects of pioglitazone and AnxA1 are independent; inflammation exerted when lacking endogenous AnxA1 could be exceedingly exacerbated, overwhelming any attempts of PPARγ-activation linked mechanisms to halt disease progression. Indeed, DSS-colitis in AnxA1^−/−^ mice is more pronounced and leads to increased mortality (Babbin et al., 2008; de Paula-Silva et al., 2016) and AnxA1 expression in the colon of WT mice increased regardless of treatment with pioglitazone. However, we here show pioglitazone prevented cleaving of AnxA1 whole 37 kDa form into smaller 33 kDa fragments, which are supposedly non-functional and induce inflammation, as evidenced in other models where AnxA1 cleaving correlates with increased inflammatory status in neutrophils (Vong et al., 2007; Williams et al., 2010; Vago et al., 2012; Sugimoto et al., 2016; Vago et al., 2016). AnxA1 can be cleaved at different cells and tissues, such as in neutrophils, adipose tissue and melanomas; non-functional 33-kDa peptides released promote inflammation facilitating cell transmigration to inflamed areas, inducing adipogenesis and causing skin tumors to become more aggressive (Williams et al., 2010; Boudhraa et al., 2014; Pietrani et al., 2018). Pioglitazone treatment also reduced MMP-9 expression in the inflamed gut of WT mice. Metalloproteinases such as MMP-9 induce inflammatory damage on tissues by cleaving extracellular matrix proteins, and that by itself can aggravate ulcerative colitis (Marshall et al., 2015; Chen et al., 2017). In this context, MMP-9 also cleaves AnxA1 into inactive 33 kDa proteins under inflammatory conditions (Zamilpa et al., 2010; Sugimoto et al., 2016). It must be noted that PPARγ expression suffered no changes due to DSS treatment or to lack of AnxA1, meaning pioglitazone could bind to its target in colon tissue at similar levels for all experimental conditions. This is in accordance with other authors who also reported PPARγ levels suffer little to no variances in colon tissue throughout the development of DSS colitis (Huang et al., 2020).

Thus, our data suggest that pioglitazone might indeed be linked to AnxA1 in order to exert its anti-inflammatory actions during the progression of DSS-induced colitis, but by modulating its functionality rather than its overall expression. Considering AnxA1 is a strong candidate for predictor of disease remission due to therapies on IBDs and AnxA1 expression levels tend to vary greatly in IBD patients, such information might aid elucidating why clinical trials investigating PPARγ ligands on IBDs tend to fail (Lewis et al., 2001; Lewis et al., 2008).

It is known that lamina propria residential macrophages play an important housekeeping role by phagocytosing apoptotic epithelial cells, clearing translocated bacteria and promoting epithelial stem cell maturation (Na et al., 2019). On the course of IBDs, residential macrophages and recruited monocytes become inflammatory M1 macrophages, which secrete inflammatory cytokines such as TNFα, IL-12, and IL-23 and induce Th1 responses (Bernardo et al., 2017; Na et al., 2019). However, during resolution phases of IBDs, lamina propria macrophages acquire a M2 phenotype and promote tissue repair due to releasing several anti-inflammatory factors such as IL-10 and AnxA1 (Na et al., 2019; Koelink et al., 2020). Macrophages are thus vital for both sustaining intestinal homeostasis and carrying intestinal inflammation, and evidence suggests AnxA1 released by these cells during resolutive phases of IBD can be responsible for attenuating disease progression (Vong et al., 2012; Quiros et al., 2017). Also, PPARγ in macrophages plays an important role on IBD physiopathology, as macrophage-specific PPARγ knockdown leads to pronounced colitis clinical effects, accumulation of TCD8+ lymphocytes in lamina propria, increase of CD40 surface expression and secretion of inflammatory cytokines in the intestine (Shah et al., 2007; Hontecillas et al., 2011). Data here obtained show tissue sections from WT mice had a basal counting of CD68^+^ macrophages which increased due to DSS administration, regardless of pioglitazone treatment, and indeed, corresponding areas on sections immunostained for AnxA1 evidenced there is a positive correlation between number of CD68^+^ macrophages and AnxA1 expression, as both increased proportionally when mice were subjected to development of DSS-induced colitis. Thus, tissue inflammation caused by DSS led to recruitment of AnxA1 secreting monocytes in order to halt inflammation and evoke tissue repair (Steinbach and Plevy, 2014; Na et al., 2019). Other authors have reported increased number of CD68^+^ macrophages in tissue sections either from biopsies specimens of Crohn’s disease or ulcerative colitis patients or from intestines of DSS-treated mice, corroborating our data (Vong et al., 2012; Hong et al., 2014; Lissner et al., 2015; Reischl et al., 2020). However, studies correlating counting of CD68^+^ macrophages with AnxA1 expression report seemingly contradicting evidence. It is reported by authors in biopsies from Crohn’s disease patients that AnxA1 expression correlates only with myeloperoxidase-positive neutrophils excluding CD68^+^ macrophages in lamina propria (Reischl et al., 2020), but others report the opposite by showing AnxA1 expression correlates with increased CD68^+^ counting in tissue biopsies from ulcerative colitis and Crohn’s disease patients, in agreement with our findings (Vong et al., 2012). Given the complexity of IBD as a disease, it is unsurprizing contradicting evidence would be found, and our work contributes in elucidating such ambiguities.

While the findings here described utilizing a murine model of experimental colitis so far evidence there is a connection between pioglitazone actions and AnxA1, assessment of AnxA1 as a marker of disease progression under pioglitazone treatment should still be considered in a preliminary manner and approached with due reservations in further studies involving humans. The immune system of mice is quite different from that of humans, and different immune responses in the gut can lead to different inflammatory processes being observed in both experimental colitis and human IBD which are not always similar, such as seen for activation of Treg cells, secretion of cytokines and infiltration of B lymphocytes (Del Prete et al., 1993; Stevceva et al., 2001; Mestas and Hughes, 2004; Gibbons and Spencer, 2011; Neurath, 2014; Wang et al., 2015; Do et al., 2017; Sun et al., 2017). Still, despite there being discrepancies, certain AnxA1 roles described in murine models of experimental colitis are corroborated in human IBD patients, such as infliximab treatment in AnxA1 knockout mice undergoing DSS colitis failing to attenuate disease progression, which links to other findings evidencing infliximab-responsive IBD patients show a greater expression of AnxA1 in colon tissue (Sena et al., 2013; de Paula-Silva et al., 2016). The same is true for PPARγ, as Crohn’s disease-like ileitis is prevented in disease-susceptible SAMP/Fc mice crossbred with disease-resistant mice due to inheritance of functional PPARγ alleles, and certain polymorphisms in the PPARγ gene have been described as prevalent in Crohn’s disease patients (Sugawara et al., 2005). In the same vein, a number of drugs used for treatment of IBD in humans have been successfully validated for use in experimental colitis models in mice (Melgar et al., 2008). Translation between mouse colitis models and human IBD is thus very feasible, but future assessment of mechanisms involving AnxA1 and pioglitazone as described in this work, when in human IBD patients, should therefore consider the aforementioned reservations.

In order to reinforce our *in vivo* data, an *in vitro* experimental protocol was carried out using the RAW 264.7 macrophage cell line. Using LPS to mimic an inflammatory environment, we found that LPS treatment increased secretion of TNFα and IL-10 and generation of ROS, effects abrogated due to treatment with pioglitazone. Corroborating our *in vivo* data, the anti-inflammatory effects of pioglitazone were only detected in mock-transfected cells, but not in AnxA1 knockdown cells, which evidences endogenous AnxA1 is vital for pioglitazone anti-inflammatory effects in macrophages. The same manner as seen in our *in vivo* data, pioglitazone did not modify AnxA1 expression. It has been reported that rosiglitazone and prostaglandin J2, both PPARγ ligands, increase AnxA1 expression, but such has been demonstrated in MDA-MB-231 and MCF-7 breast cancer cells to variable results and not at inflammatory conditions (Chen et al., 2017). LPS also increased AnxA1 expression by RAW 264.7 cells and expressed AnxA1 was cleaved into smaller, non-functional 33 kDa fragments, and regardless of the effects of pioglitazone on AnxA1 expression, it prevented AnxA1 cleaving and attenuated secretion of MMP-9. Control of MMP-9 secretion is a known anti-inflammatory effect of pioglitazone and other PPARγ ligands; they can modulate septin-2 in cancer cells preventing MMP-9 actions, and during the course of intestinal inflammations, down-modulation of PPARγ can make MMP-9 more stable (Kundu et al., 2014; Cao et al., 2015). Still, it had not yet been demonstrated that such effect could be linked to prevention of AnxA1 cleaving. As described previously, pioglitazone seems to attenuate inflammation by preventing AnxA1 cleaving rather than controlling its overall expression, and using our *in vitro* model we found evidence that so happens in macrophages, strengthening our *in vivo* data. Of note, contrary to what was seen *in vivo*, inflammatory conditions increased PPARγ expression in mock-transfected but not in AnxA1-knockdown macrophages. Under non-inflammatory conditions, pioglitazone by itself induced PPARγ expression, as expected due to it being a PPARγ ligand, but also only in mock-transfected cells. It is reported LPS activates PPARγ as a means of “desensitizing” macrophages attenuating responses to further inflammatory stimuli, and AnxA1 seems to be involved with such modulation of PPARγ in both inflammatory and non-inflammatory conditions (Von Knethen and Bernhard, 2001). Whether lack of AnxA1 prevents pioglitazone binding to PPARγ or prevents some sort of post-translational mechanism after interaction between PPARγ and a ligand was not assessed in our work, but the fact this interaction was seen in isolated macrophage cultures and not in mice tissue further reinforces the need for *in vitro* models to better investigate specific effects which might become hidden amid the myriad of simultaneous biological processes occurring *in vivo*. Indeed, PPARγ in colon tissue is expressed not only by lamina propria macrophages, but also at considerable levels by crypt epithelial cells (Su et al., 2007).

While pioglitazone showed its anti-inflammatory effects to be reliant on AnxA1, it is known pioglitazone has other effects that are PPARγ-independent, such as induction of migration of vascular smooth muscle cells and inhibition of proliferation of tumor cells (Emery et al., 2006; Li et al., 2008). By expanding some of the *in vitro* findings previously described, we evidenced some of the anti-inflammatory actions of pioglitazone are indeed PPARγ-dependent, as treatment of RAW 264.7 cells with the PPARγ antagonist GW9662 prevented the increased secretions of TNFα and MMP-9 to be attenuated by pioglitazone. To our knowledge, this effect has been previously described for TNFα in myocytes, but not in RAW 264.7 macrophages (Liu et al., 2009). This is relevant for prospective studies on PPARγ ligands and AnxA1, as knowing that inhibition of MMP-9 secretion is dependent on PPARγ makes it more likely that other PPARγ ligands studied for treatment of IBD would have the same effect in preventing AnxA1 cleaving.

As to further elucidate how AnxA1 could influence the anti-inflammatory effects of pioglitazone, we investigated whether AnxA1 knockdown of RAW 264.7 macrophages could affect pioglitazone-induced phosphorylation of extracellular regulator kinase (ERK). ERK is a signaling factor linked to several cell processes, such as cell survival, differentiation and migration, among others (Maurice et al., 2018; Li et al., 2019; Zhou et al., 2019), and it is known that pioglitazone can activate ERK1/2 phosphorylation leading to anti-inflammatory effects, as reported on ischemic cardiomyocytes and on different cell lines, including colon cell lines (Wang et al., 2012; Kole et al., 2016). Our data showed that pioglitazone did cause ERK phosphorylation, but such effect was not seen in AnxA1 knockdown cells, evidencing AnxA1 is required for pioglitazone-induced ERK phosphorylation to occur. ERK phosphorylation is also known to take place *downstream* to AnxA1 activation in a plethora of cells, including macrophages (Dufton et al., 2010; Bena et al., 2012). As we evidenced lacking AnxA1 prevents ERK phosphorylation, it can be inferred that AnxA1 might be the sole activator of ERK phosphorylation *downstream* to PPARγ activation in this scenario, being an intermediate molecule connecting pioglitazone actions and ERK phosphorylation, which is allowed to activate ERK by pioglitazone by remaining functional and non-cleaved for longer. In addition, increased FPR2 expression, but not FPR1, in AnxA1-knockdown cells suggests there is a cell response under absence of AnxA1 which aims to increase FPR2-mediated downstream signaling, which is known to involve ERK phosphorylation (Bena et al., 2012) leading to most anti-inflammatory effects of AnxA1, such as reduction of inflammatory cytokines and of neutrophil activation in different murine models of inflammation (Ding et al., 2020; Machado et al., 2020). Pioglitazone, by preserving full length-AnxA1, thus possibly increases the likelihood for AnxA1 to bind to FPR2 leading to ERK phosphorylation.

**FIGURE 9:**
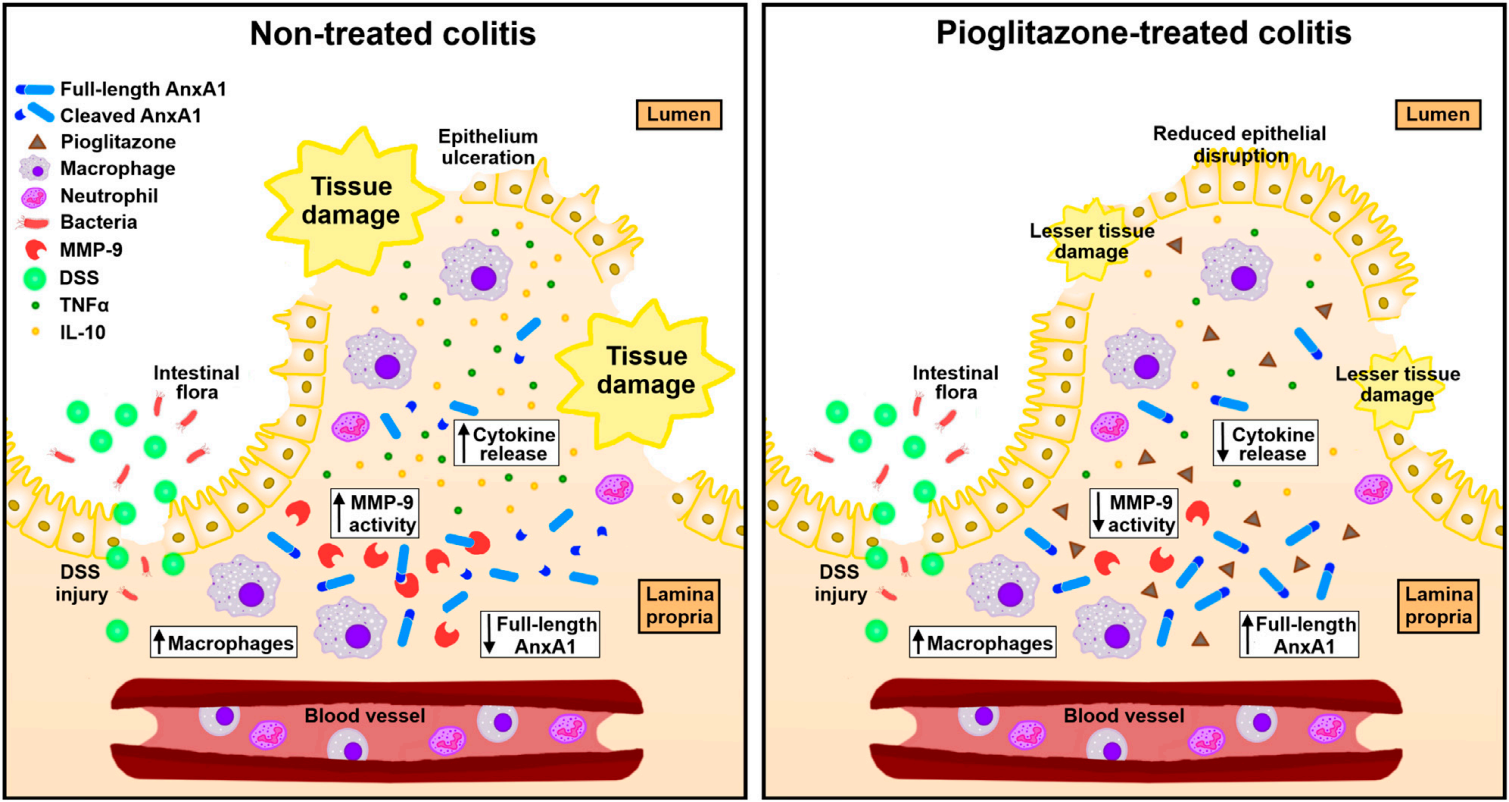
Schematics of connections between pioglitazone effects and AnxA1 on experimental colitis. In experimental colitis induced by DSS, tissue injury exposes intestinal lamina propria to gut microorganisms triggering inflammation and increasing the number of AnxA1-releasing macrophages. AnxA1 released this way is cleaved into smaller, non-functional fragments by proteolytic enzymes such as MMP-9, which impairs its anti-inflammatory actions leading to greater secretion of cytokines such as TNFα and IL-10 and causing overall disruption of the intestinal tissue. Upon treatment with pioglitazone, MMP-9 expression decreases and causes AnxA1 released by macrophages to remain non-fragmented for longer, allowing it to promote its anti-inflammatory actions, decreasing cytokine secretion and reducing overall tissue disruption.

Overall, our main findings evidence pioglitazone relies on AnxA1 expressed by macrophages to exert its anti-inflammatory actions throughout the course of experimental colitis, and that it does so by preventing AnxA1 cleaving. A graphical representation of such findings is depicted in Figure 9.

## Conclusion

Data obtained in the present work evidence pioglitazone attenuates inflammation *in vivo* in a murine colitis model, and prevention of AnxA1 cleaving seems to be involved with such effect. Data obtained *in vitro* show that this process occurs in macrophages, and that pioglitazone effects rely on ERK phosphorylation, which requires AnxA1. This sum of evidence allows us to suggest that the beneficial effects of pioglitazone on the treatment of IBDs are connected to AnxA1 cleaving rather than its expression. As AnxA1 has been increasingly targeted as a potential predictor of IBD progression and treatment-induced remission, this evidence is of value for future clinical trials investigating PPARγ ligands for treatment of IBDs, as biomarkers which might indicate more accurately how therapeutic success on patients is more likely to be achieved, such as AnxA1, can be more thoroughly assessed.

## Data Availability Statement

The raw data supporting the conclusions of this article will be made available by the authors, without undue reservation.

## Ethics Statement

The animal study was reviewed and approved by Ethics Committee on Animal Use of the Faculty of Pharmaceutical Sciences, University of São Paulo.

## Author Contributions

GdR and SF designed the study and wrote the final manuscript. GdR, MdP-S, MB, PS, and LM performed experiments. LM and SM-E provided expertize on cell transfection protocols.

## Funding

This work received financial support from the National Council for Scientific and Technological Development (CNPq, no. 130090/2019‐0) and from the São Paulo Research Foundation (FAPESP, nos. 2014/07328‐4, 2016/19682-2, 2017/05430‐4 and 2018/26383‐7), which also provided funds for open access publication fees.

## Conflict of Interest

The authors declare that the research was conducted in the absence of any commercial or financial relationships that could be construed as a potential conflict of interest.
