# Supplementary figures and images for: Pioglitazone-Mediated Attenuation of Experimental Colitis Relies on Cleaving of Annexin A1 Released by Macrophages
Source: Front Pharmacol. 2020 Dec 21;11:591561. doi: 10.3389/fphar.2020.591561 (PMC7845455; doi:10.3389/fphar.2020.591561)

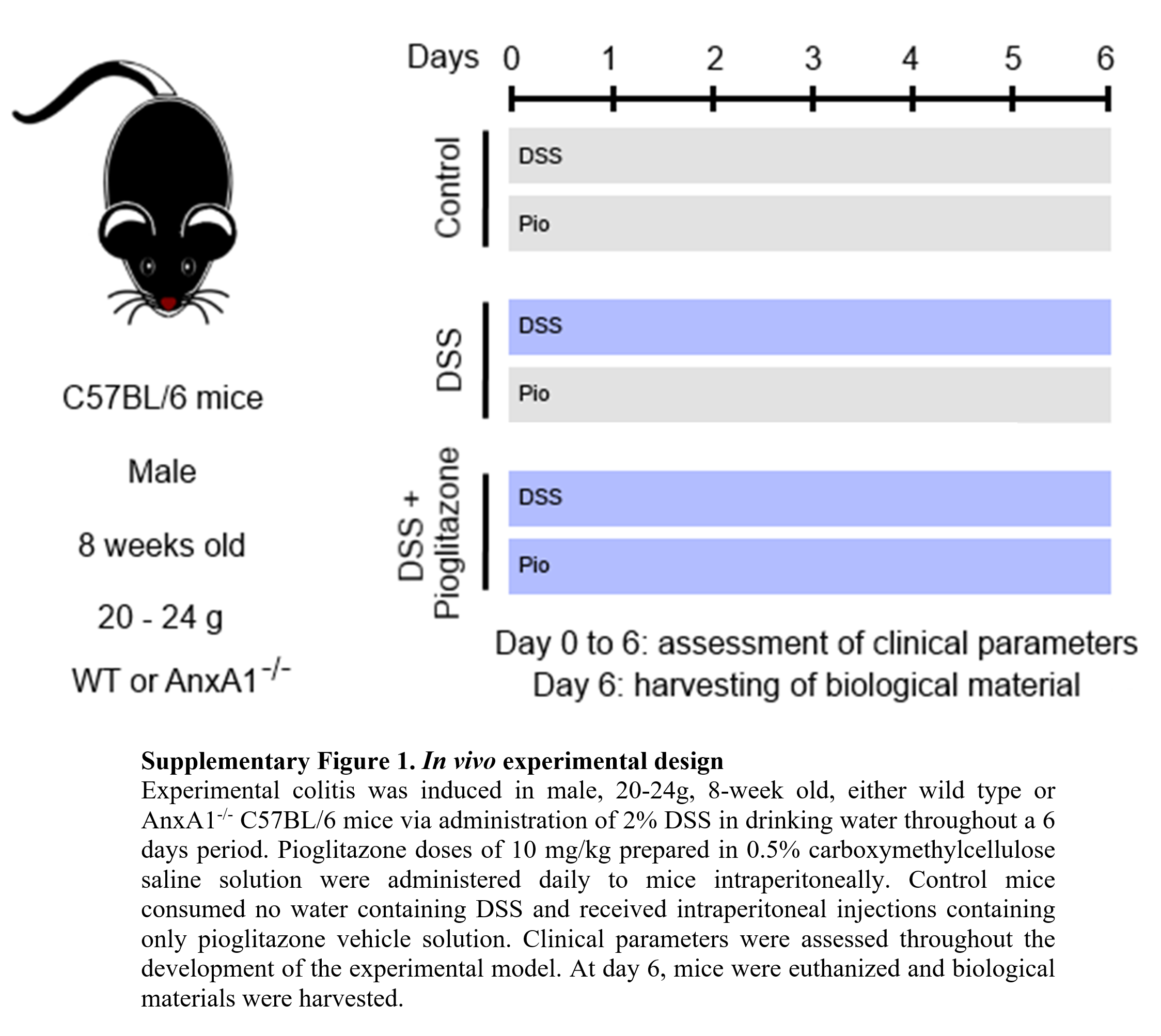

Supplement: Supplementary file 1 [file image1.tif]

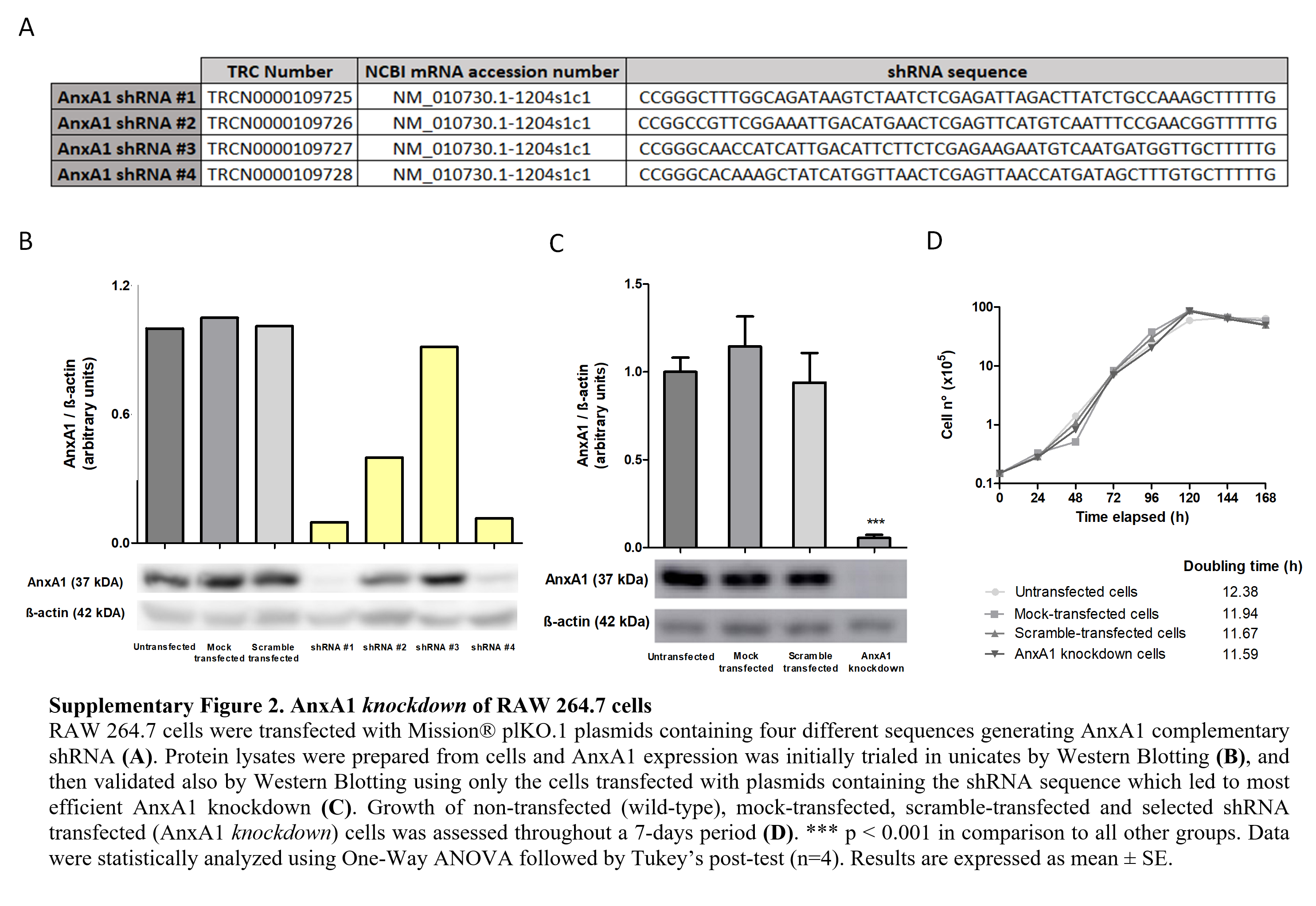

Supplement: Supplementary file 2 [file image2.tif]

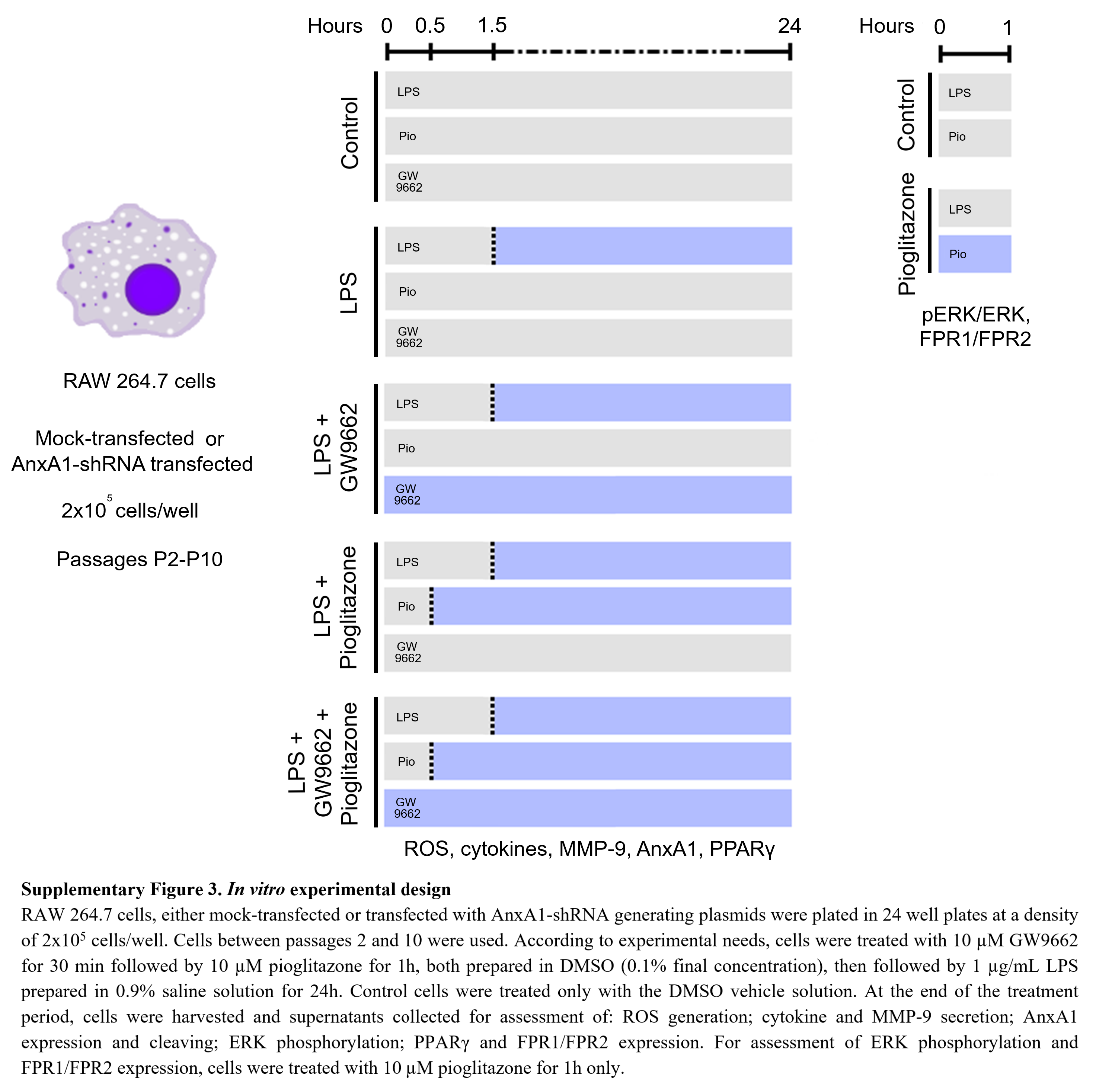

Supplement: Supplementary file 3 [file image3.tif]

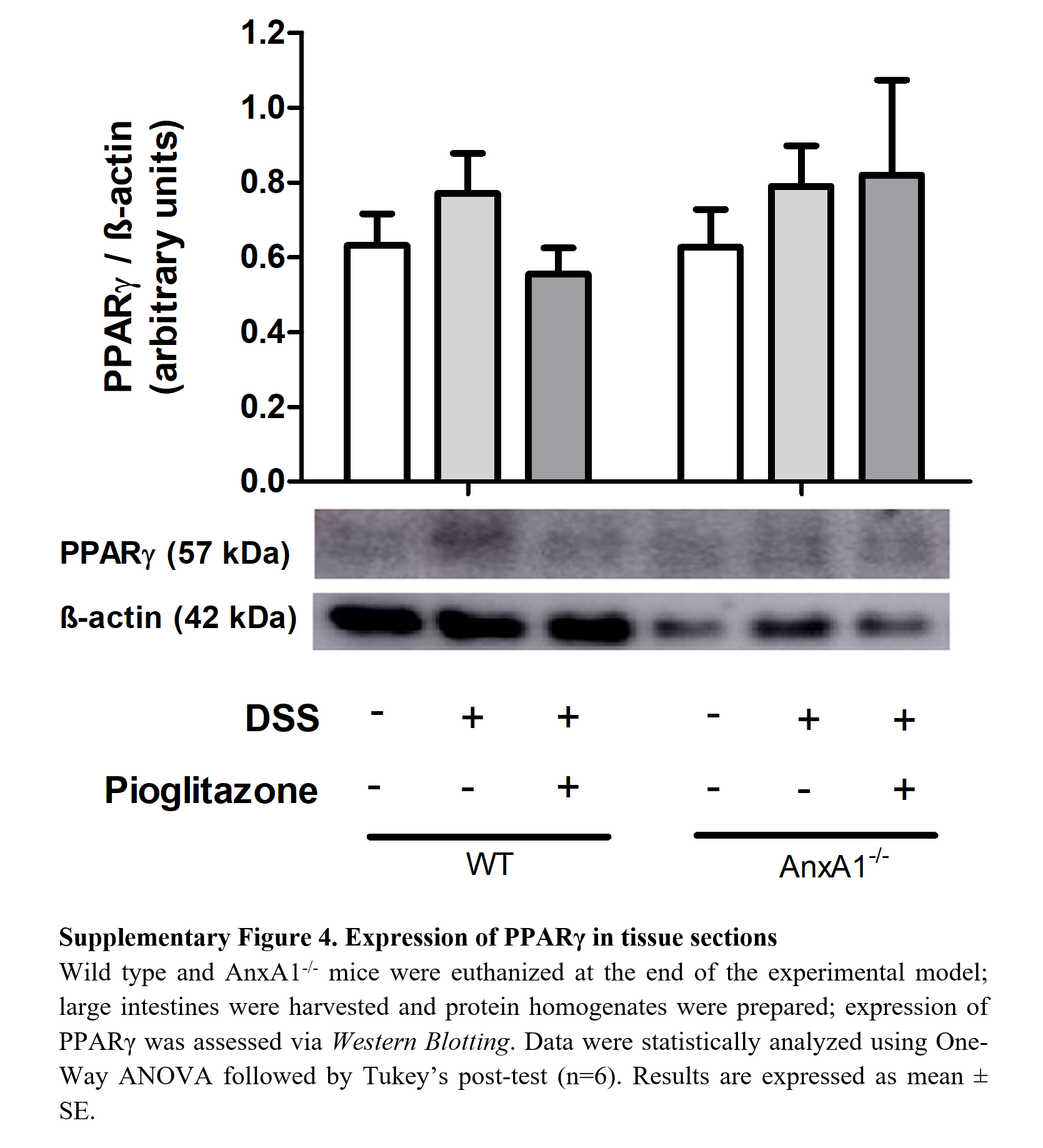

Supplement: Supplementary file 4 [file image4.tif]

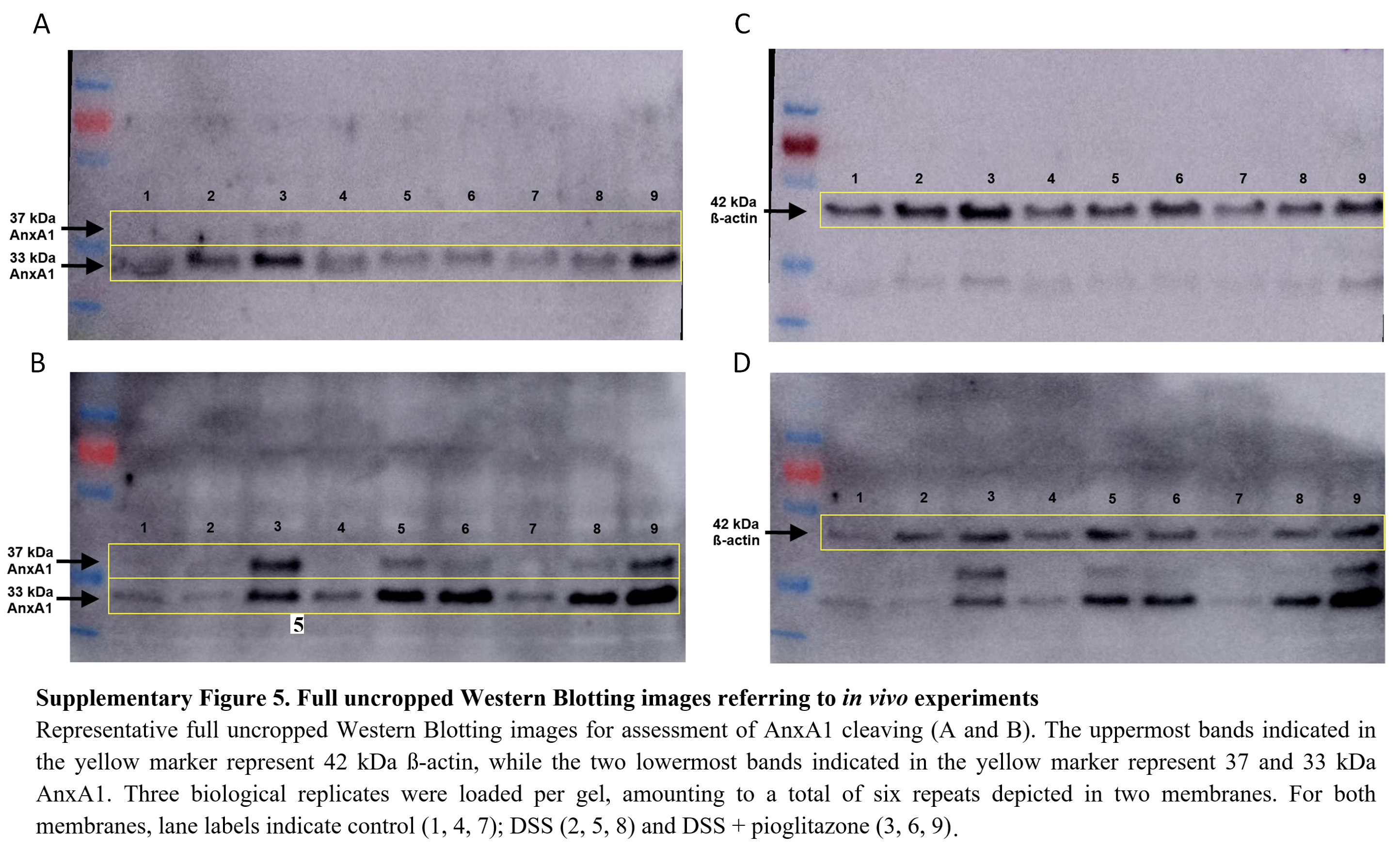

Supplement: Supplementary file 5 [file image5.tif]

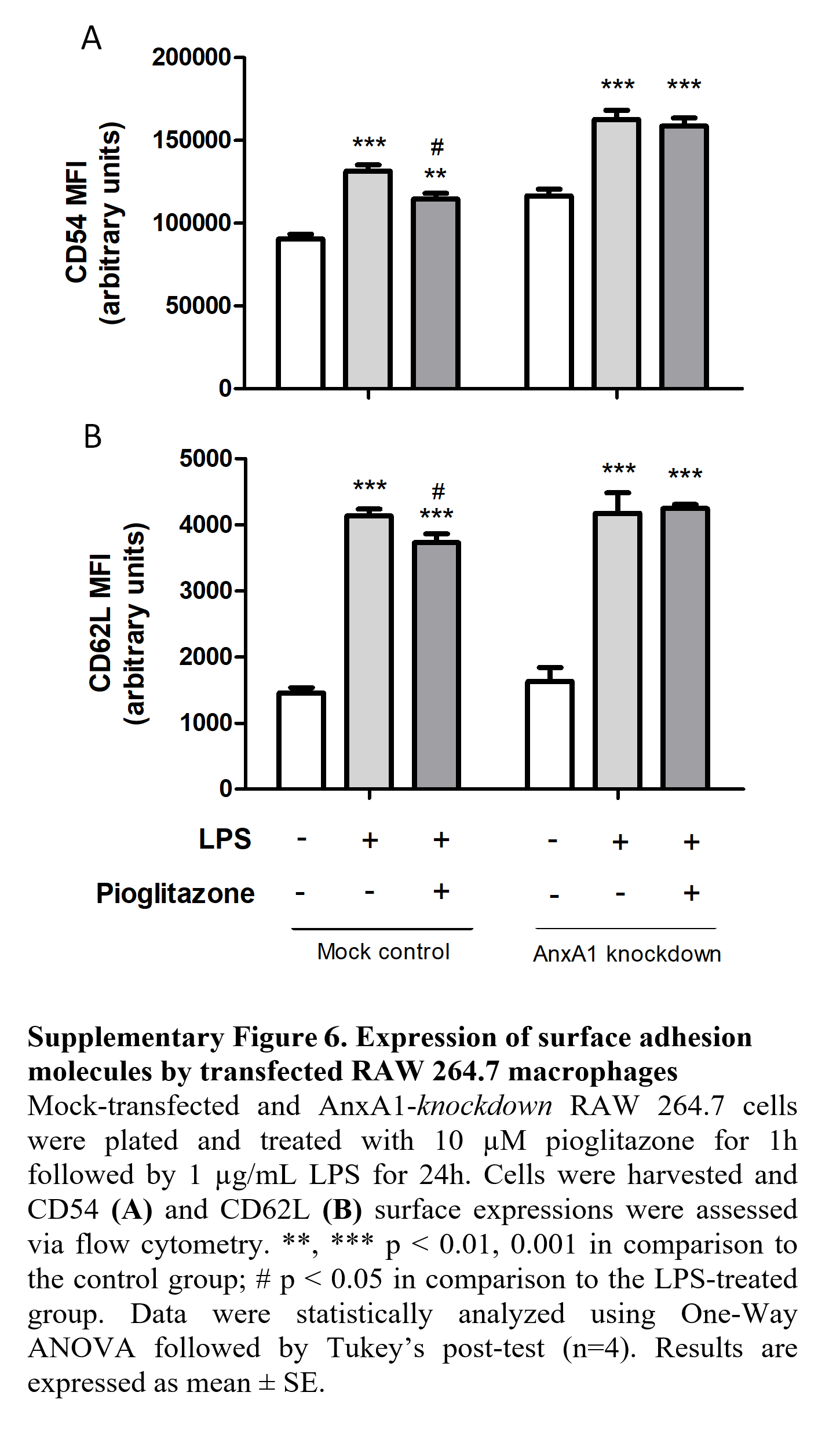

Supplement: Supplementary file 6 [file image6.tif]

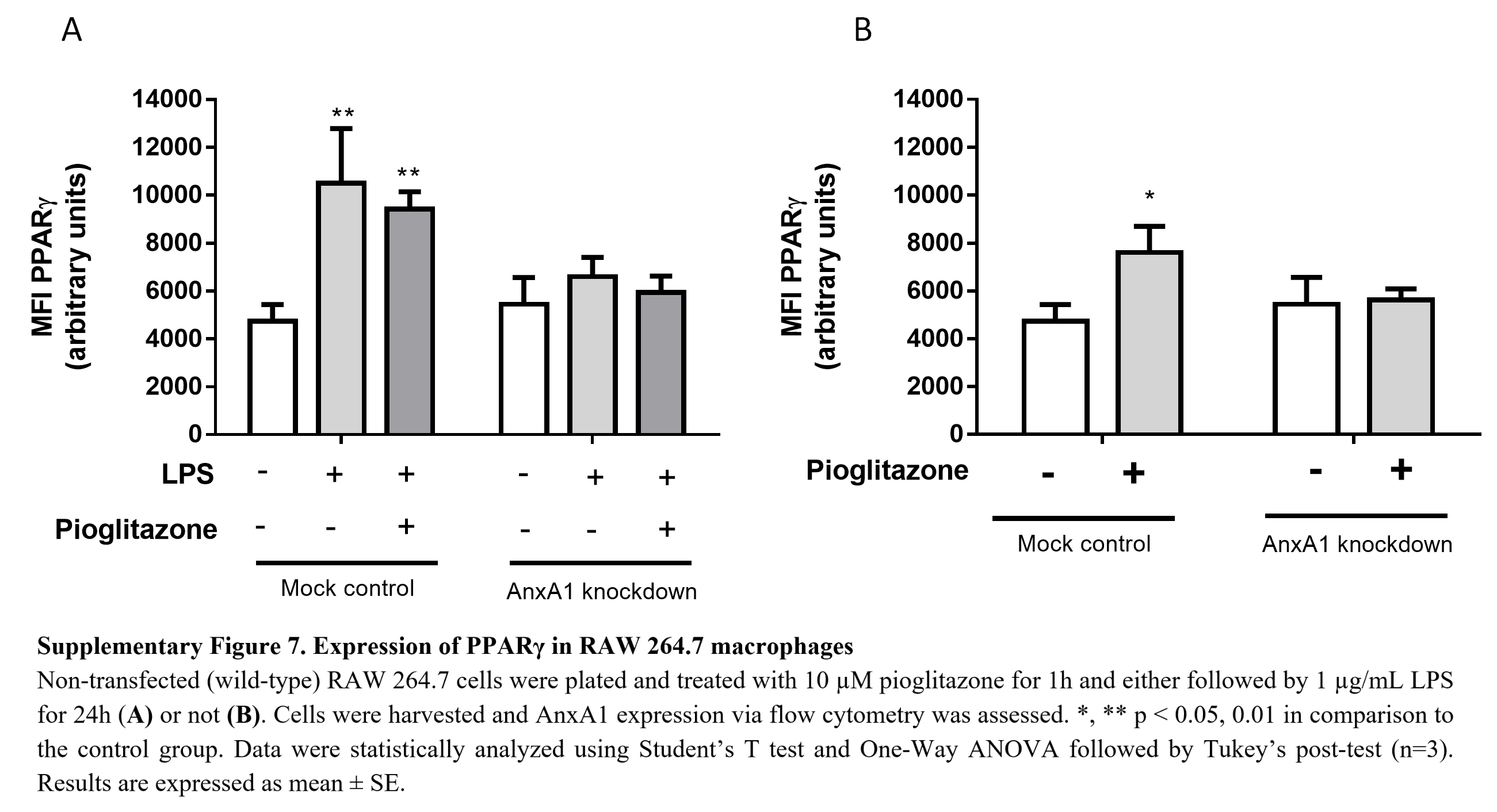

Supplement: Supplementary file 7 [file image7.tif]

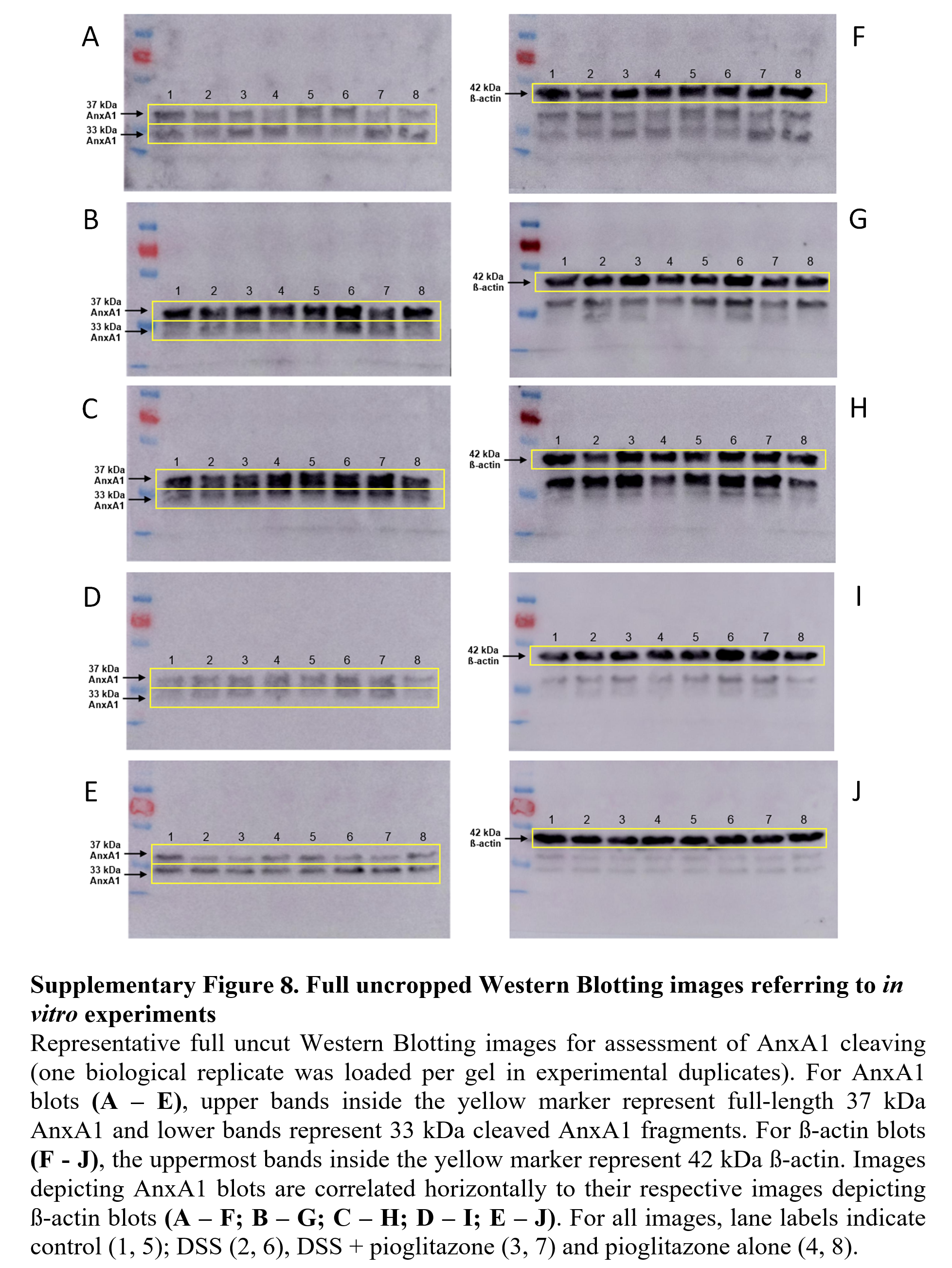

Supplement: Supplementary file 8 [file image8.tif]

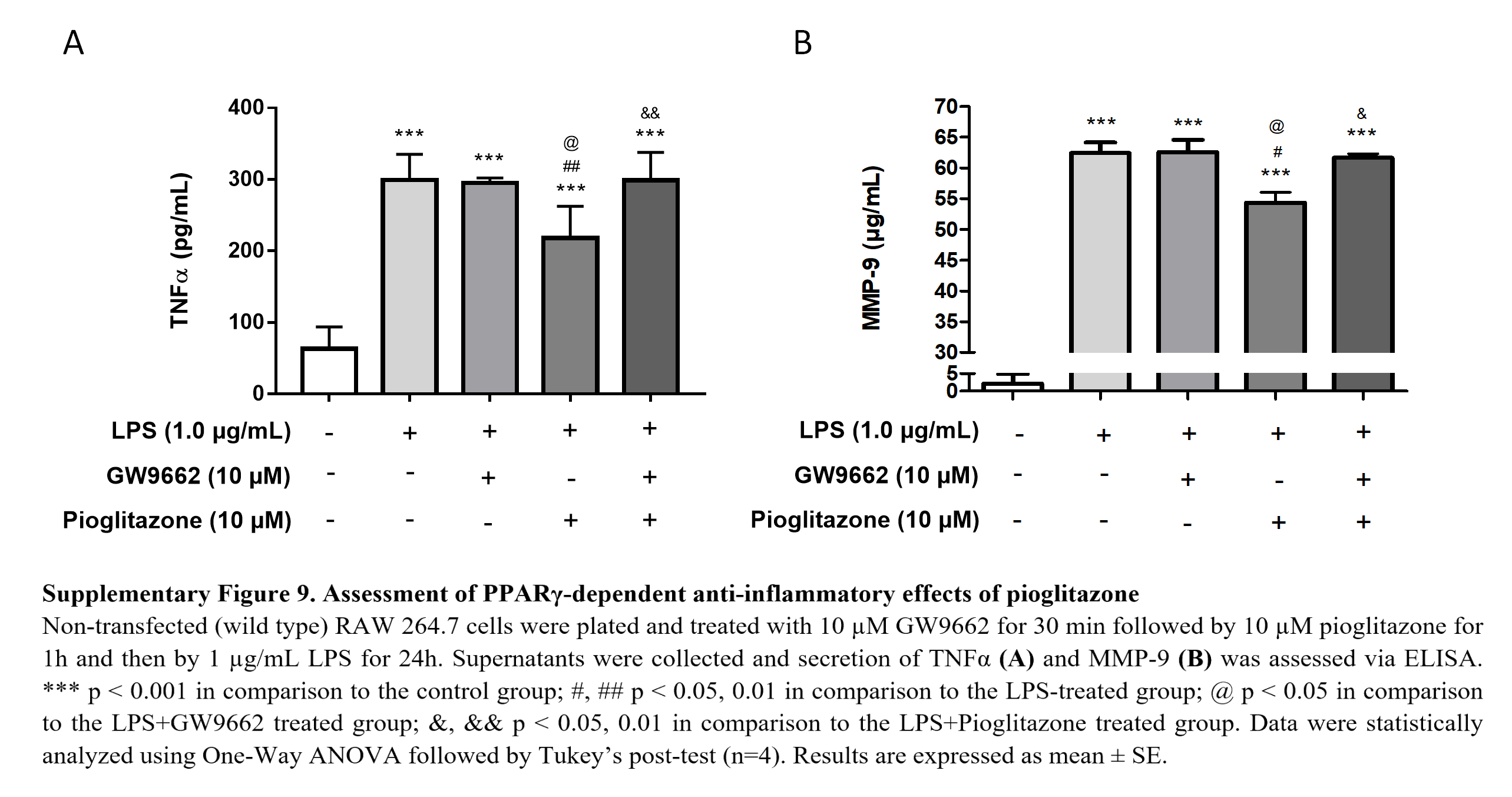

Supplement: Supplementary file 9 [file image9.tif]
